# Two β-Lactamase Variants with Reduced Clavulanic Acid Inhibition Display Different Millisecond Dynamics

**DOI:** 10.1128/AAC.02628-20

**Published:** 2021-07-16

**Authors:** Wouter Elings, Aleksandra Chikunova, Danny B. van Zanten, Ralphe Drenth, Misbha Ud Din Ahmad, Anneloes J. Blok, Monika Timmer, Anastassis Perrakis, Marcellus Ubbink

**Affiliations:** a Leiden Institute of Chemistry, Leiden University, Leiden, The Netherlands; b Division of Biochemistry, the Netherlands Cancer Institute, Amsterdam, The Netherlands; c Oncode Institute, Amsterdam, The Netherlands

**Keywords:** BlaC, NMR spectroscopy, inhibition, chemical exchange, directed evolution, error-prone PCR, X-ray crystallography

## Abstract

The β-lactamase of Mycobacterium tuberculosis, BlaC, is susceptible to inhibition by clavulanic acid. The ability of this enzyme to escape inhibition through mutation was probed using error-prone PCR combined with functional screening in Escherichia coli. The variant that was found to confer the most inhibitor resistance, K234R, as well as variant G132N that was found previously were characterized using X-ray crystallography and nuclear magnetic resonance (NMR) relaxation experiments to probe structural and dynamic properties. The G132N mutant exists in solution in two almost equally populated conformations that exchange with a rate of ca. 88 s^−1^. The conformational change affects a broad region of the enzyme. The crystal structure reveals that the Asn132 side chain forces the peptide bond between Ser104 and Ile105 in a *cis*-conformation. The crystal structure suggests multiple conformations for several side chains (e.g., Ser104 and Ser130) and a short loop (positions 214 to 216). In the K234R mutant, the active-site dynamics are significantly diminished with respect to the wild-type enzyme. These results show that multiple evolutionary routes are available to increase inhibitor resistance in BlaC and that active-site dynamics on the millisecond time scale are not required for catalytic function.

## INTRODUCTION

**FIG 1:**
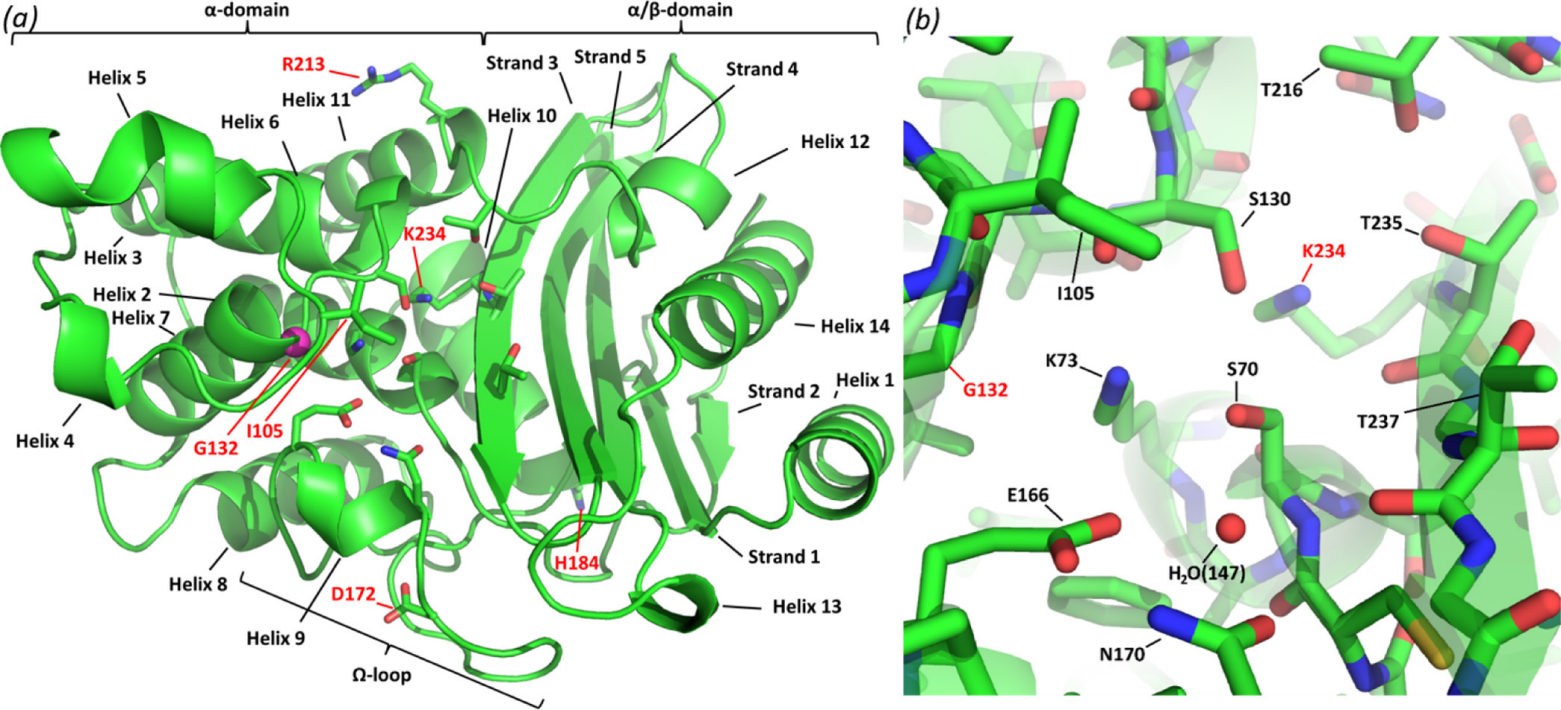
Structure of BlaC (chain A under PDB accession number 5NJ2 [26]). (a) BlaC in a cartoon representation, with several relevant residues in a stick representation and the C_α_ of Gly132 in a magenta sphere representation. Domains, α-helices, β-strands, and the Ω-loop are indicated in black, while the residues that were mutated in this study are indicated in red. (b) Detail of the active site showing both a stick representation and a transparent cartoon representation. Several active-site residues and the conserved active-site water molecule are indicated, of which the main topics of this study, G132 and K234, are in red. (Adapted from reference 10.)

Chemical reactions are catalyzed by enzymes through stabilization of the transition state, by arranging functional groups in the active site in precise orientations with respect to the substrate. Some enzymes have broad substrate profiles, catalyzing reactions with a variety of substrates. As different substrates can have different transition states, such enzymes must have some degree of flexibility in the active site. Furthermore, in some cases, a single-amino-acid mutation can have a profound effect on function, switching the specificity of an enzyme from one substrate to another (e.g., see references 1–3). Conformational dynamics are required for protein evolution, as flexibility allows promiscuity and adaptation by single-amino-acid substitutions (4–9). We have recently characterized the dynamic behavior of the β-lactamase of Mycobacterium tuberculosis, BlaC (Fig. 1), and shown that this enzyme indeed features pronounced millisecond dynamics around its active site (10). It can be hypothesized that apart from allowing the enzyme to hydrolyze its extraordinarily broad spectrum of substrates, this flexibility may aid the enzyme to evolve more readily to gain new functionalities. Evolutionary constraints of BlaC include factors such as efficient folding and export, stability at 37°C, and the ability to break down a wide range of β-lactams. Such environmental constraints may change over time, requiring the protein to adapt. With the increasing clinical use of β-lactam/β-lactamase inhibitor combinations for the treatment of tuberculosis, the extent to which BlaC is able to adapt to evade inhibition will become especially interesting. Two single-amino-acid variants of BlaC that exhibit reduced inhibition by the clinically most used β-lactamase inhibitor, clavulanic acid, are the subject of this work.

One of the most conserved motifs in class A β-lactamase active sites is the serine-aspartate-asparagine (SDN) motif at Ambler positions 130 to 132. In BlaC, however, the asparagine at position 132 is replaced by a glycine. This substitution effectively removes a side chain with an amide functional group from the active-site pocket, resulting in an enlarged active site in BlaC. This wide active site has been suggested to be important for the broad substrate profile of BlaC. Specifically, substituents at the R^6^ position of carbapenem could be accommodated by this substitution (11). Soroka et al. investigated the effect of a G132N mutation in BlaC (3, 12). The impact of the substitution on the hydrolysis rate differs per substrate. In BlaC, G132N increases the hydrolysis rate of nitrocefin, imipenem, and aztreonam while decreasing that of cefoxitin and ceftazidime. Interestingly, however, this single mutation was found to enable BlaC to hydrolyze clavulanic acid while simultaneously increasing the efficiency of inhibition by another inhibitor, avibactam. For BlaC G132N and the wild type (wt), the same adduct masses were observed upon reaction with clavulanate, so the increased turnover rate in the former must represent either impaired tautomerization or increased hydrolysis of the tautomers. Analogously, in the class A β-lactamases KPC-2, CTX-M-1, and BlaMab, which have an Asn residue in this position, the mutation to Gly leads to a reduced affinity for avibactam and increased stability of acyl-enzyme complex with clavulanate (3, 12, 13). On the other hand, inhibition by clavulanate has been reported for many SDN-containing β-lactamases (14–16), so the effect of this substitution appears to be context specific. Multiple mycobacterial β-lactamases occur in both groups.

Another conserved motif in class A β-lactamase active sites is the KTG motif at Ambler positions 234 to 236 in the carboxylate-binding pocket. This motif occurs in almost all class A β-lactamases, including BlaC, but not in the carbenicillin-hydrolyzing enzymes, where it is replaced by an RTG motif. The amine of Lys234 is nested in the wall of the carboxylate-binding pocket, where its charge plays an important role in electrostatic interactions with the substrate. Furthermore, mutational studies have shown that it is also involved in transition state stabilization (17). The mutation K234R was found to increase resistance to clavulanic acid in class A β-lactamases such as SHV-1 (18), SHV-72 (19), and SHV-84 (20), in most cases without significantly decreasing catalytic activities against penicillins. Interestingly, in another class A β-lactamase, KPC-2, the K234R mutation did not show enhanced resistance against inhibition by clavulanic acid, but it showed enhanced resistance against inhibition by avibactam (21). Egesborg et al. found that this mutation also confers resistance to clavulanic acid in BlaC (20). The resistance to inhibition by clavulanic acid was shown to increase further in BlaC by combining K234R with the S130G or R220S mutation, although these mutations significantly decrease the enzymatic activity. Here, we report a laboratory evolution experiment aimed at finding mutants that have increased resistance against inhibition with clavulanic acid. The most prominent one found was the previously reported K234R mutation. To understand the effects of this mutation and G132N on the dynamics of the enzyme, nuclear magnetic resonance (NMR) relaxation analysis was performed, and the structure of G132N BlaC was solved. Remarkable changes in dynamics in comparison to the wt enzyme were observed. BlaC G132N shows enhanced dynamics and occurs in two states that are nearly equally populated, whereas in BlaC K234R, the millisecond dynamics observed in the active site of wt BlaC is lacking. Possible relationships between dynamics and function are discussed.

## RESULTS

**FIG 2:**
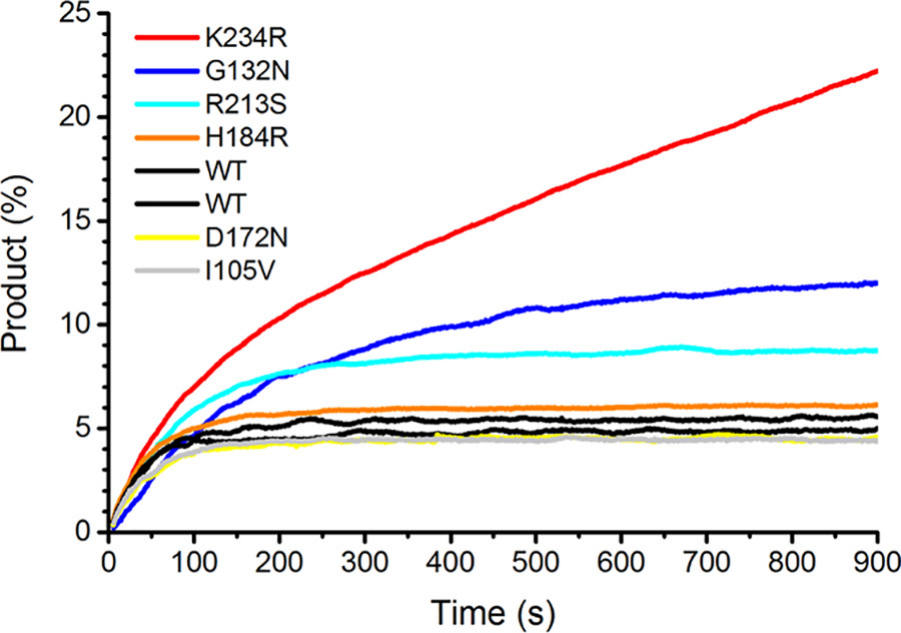
Inhibition curves. The product formation of nitrocefin hydrolysis in time in the presence of clavulanic acid (10 μM) is plotted for wild-type (WT) BlaC and single-amino-acid mutants. The product is displayed as a fraction of the product that is converted by the BlaC variant in 900 s, in the absence of an inhibitor.

A library of BlaC mutants was generated, and approximately two million CFU were screened for their ability to provide Escherichia coli with increased resistance to ampicillin and clavulanic acid, relative to wt BlaC. Several mutants were found, most of them carrying multiple mutations. To test if the increased resistance could be attributed to any single mutation, single-amino-acid mutants were prepared from those mutants that conveyed the most resistance to E. coli. The most promising amino acid mutations were I105V, D172N, H184R, R213S, and K234R. Mutations D172N, R213S, and K234R were found together in one clone, as were H184R and K234R. I105V was found in combination with D42G and R277C, which were not further tested as single mutants. The mutation G132N was not found, perhaps because it requires at least two mutations in the codon for G132 (GGC>AAC) and, thus, is less likely to occur. The corresponding single-amino-acid mutant proteins, with the addition of the G132N mutant, were produced and purified. Their respective abilities to resist inhibition by clavulanic acid were characterized by measuring the onset of inhibition by 10 μM clavulanic acid (Fig. 2). The G132N and K234R mutations convey resistance to inhibition, maintaining some level of activity even after prolonged exposure. The R213S mutant was found to require more time than wt BlaC to reach full inhibition. It is not obvious why, as this side chain is not located close to the active site but rather is located on the outside of the protein, interacting mostly with the solvent. This mutant was not further investigated. The effects of the I105V, D172N, and H184R mutations on inhibition were not significant in this assay. The reason why I105V was found in our screen may be a slightly increased ampicillinase activity due to a widening of the active site, as was discussed previously by Feiler et al. (22). The other mutations for which no effect on inhibition was observed were found in combination with the K234R mutation. Their effects may be epistatic or stability related, but the more obvious explanation would be that the mutations are random, without any functional effect. These variants were not investigated further.

**FIG 3:**
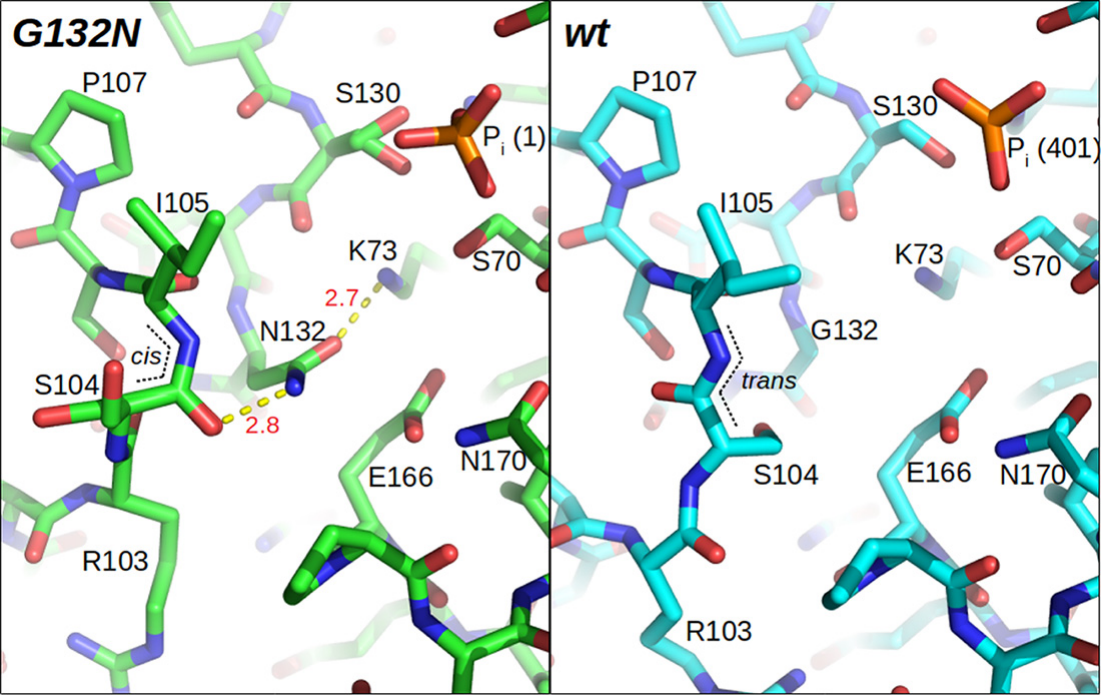
Active sites of the BlaC G132N (in green) and wt (chain A under PDB accession number 5NJ2) (in cyan) (26) crystal structures. The Asn132 side chain hydrogen bonds are indicated with yellow dashed lines, with distances in angstroms indicated in red. The *trans*- to *cis*-peptide bond isomerization is indicated with black dotted lines.

As discussed above, the effects of K234R and G132N mutations in BlaC have been identified by others, and these mutants have been characterized kinetically (3, 12, 20). However, an understanding of how these mutations impact the protein structure and dynamics is still lacking. Both of these mutations insert a relatively large functional group in or near the active site, which will require an adaptation in the conformation of nearby structural elements. To elucidate the effect of the mutations on the ground state structure of BlaC, crystallization of the two purified mutant enzymes was attempted. BlaC K234R did not yield any usable crystals despite several rounds of buffer optimization and seeding. BlaC G132N gave crystals useful for X-ray diffraction, resulting in a structure at a 1.6-Å resolution and *R*_work_/*R*_free_ factor values of 15.5/18.8% (see Table S2 in the supplemental material). The structure is overall similar to that of wt BlaC, but notable differences exist (Fig. 3). The Asn side chain that is introduced at position 132 occupies the canonical position for class A β-lactamases (e.g., in TEM-1 [23] and SHV-1 [24]). The oxygen of the side chain is hydrogen bonded (2.7 Å) with the amide of Lys73. Strikingly, however, the nitrogen of the Asn132 side chain hydrogen bonds (2.8 Å) to the carbonyl oxygen of residue Ser104. This bond flips the carbonyl of Ser104 and forces it into a *cis*-peptide conformation. This is notable as this is the only *cis*-peptide conformation in this position compared to all 94 homologous structures in the Protein Data Bank (PDB), as revealed by the LAHMA server (25). This interaction in turn flips the side chain of Ser104 from the inside to the outside of the protein, where it adopts two conformations. This change propagates to residues 102 to 105, which adopt a conformation distinct from that of wt BlaC.

An interesting double conformation is also observed for the Ser130 side chain (Fig. S4a). This side chain in the BlaC G132N structure has roughly 50% occupation of the canonical position that is observed in all but six homologues in the PDB, donating a hydrogen toward a bond to the conserved active-site phosphate (2.6 Å) and perhaps receiving a hydrogen from the Lys234 amide (2.9 Å). In the second conformation, both hydrogen bonds are maintained, with the bond to the Lys234 amide becoming shorter (2.5 Å). Multiple conformations are also supported by the electron density for the Asn214, Thr215, and Thr216 loop region, two of which were modeled (Fig. S4b). In this case, the occupancy of each modeled conformation is not equal, and current modeling can be considered only an estimate indicative of the conformational dynamics.

**FIG 4:**
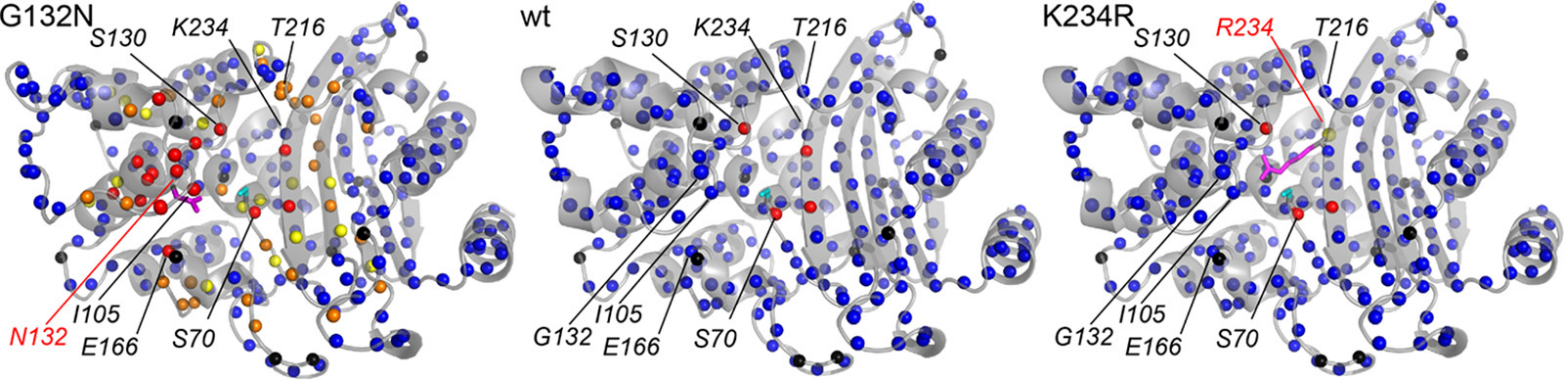
Line-broadening effects. Residues for which the backbone amide resonances were lost, split, or diminished in the spectra are color-coded on the backbone amide nitrogen atoms, indicated as spheres, for G132N (left), wt (middle), and K234R (right) BlaC. Red, no resonance could be assigned; orange, two resonances could be assigned; yellow, one resonance could be assigned, but the peak intensity diminished below half of the wt relative peak intensity; blue, one resonance could be assigned, with a normal peak intensity; black, proline. The Ser70 side chain is indicated as a cyan stick, and mutant side chains are indicated as magenta sticks, in the structure of BlaC G132N reported in this work (PDB accession number 7A74) or modeled into chain A of the wt BlaC structure (PBD accession number 5NJ2) (26).

The multiple conformations for BlaC G132N could at least partially be propagated through Ser104-Ile105 peptide bond isomerization and are suggestive of increased dynamics relative to the wild-type enzyme. We recently reported dynamics in the active site of BlaC, both in the resting state and when bound to clavulanic acid or avibactam adducts (10). We wondered whether the dynamic behavior was affected by the G132N and K234R mutations. Therefore, the ^1^H-^15^N correlation spectra of BlaC mutants G132N and K234R were assigned using HNCa spectra in combination with the assignments for wt BlaC, for which peaks have been assigned to all nonproline backbone amides except Asp2 and 4 residues in the active site, close to the phosphate-binding site (26). The mutant proteins exhibited well-dispersed spectra that show a significant overlap with the spectrum of wt BlaC, confirming that they share the same overall fold (Fig. S5). In the spectrum of BlaC K234R, peaks were assigned to all the backbone amide groups that were also assigned in wt BlaC, including Arg234. Also, the resonance belonging to the backbone amide of Thr235 could be assigned. This resonance is not detectable in the spectrum of wt BlaC (Fig. S5c, marked with *). In the spectrum of BlaC G132N, many resonances could not be detected. The region for which peaks of backbone amides have broadened beyond detection was found to have extended from the 4 residues in wt BlaC to include Glu166, the active-site-covering loop ranging from Ser104 to Val108, and the loop between helices 6 and 7, containing Ser130 and the variant residue Asn132 (Fig. 4). Surprisingly, this last stretch of undetectable amides extends all the way until Leu137, spanning half of helix 7. Furthermore, the resonances of several surrounding residues were observed to have split between two positions. These observations indicate the presence of one or more chemical exchange processes in the solution state. The split peaks have minimum distances of ∼21 and ∼24 Hz in the ^1^H and ^15^N dimensions, respectively, which means that the rate of exchange between the two states must be lower than ca. 100 s^−1^. The relative peak intensities of the split peaks indicate populations of ca. 60% and 40%. The amides for which resonances were broadened or split are centered around the G132N mutation and the rest of the active site (Fig. 4).

**FIG 5:**
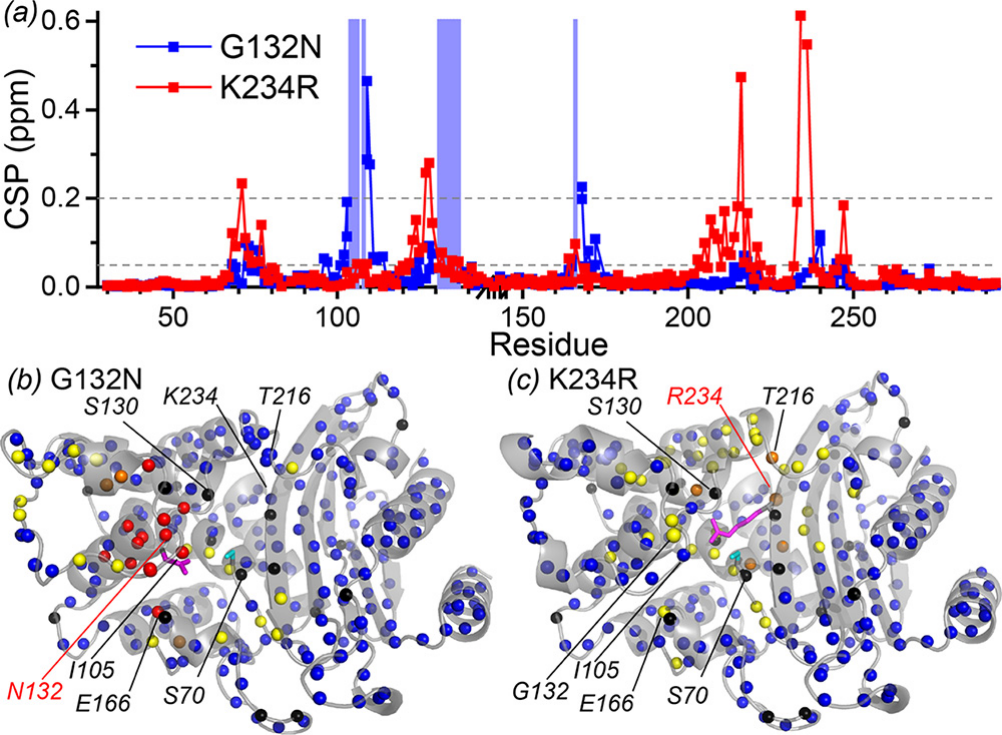
Chemical shift perturbations of BlaC backbone amide resonances upon mutation. (a) Plot of average absolute CSPs on the sequence [|1/2Δδ(^1^H)| + |0.1 × Δδ(^15^N)|]. Blue background indicates residues that have broadened beyond detection in the G132N mutant. Gray dashed lines represent the cutoff values that were used for the coloring of amides in panels b and c. The break on the horizontal axis represents the additional BlaC G-G-G-T loop, relative to Ambler numbering. Error bars have been omitted for clarity, and the estimated 95% confidence interval is ±0.03 ppm. (b and c) Plots of CSPs for BlaC G132N (b) and BlaC K234R (c) on the structure (PBD accession numbers 7A74 for BlaC G132N and 5NJ2, chain A [26], for the model K234R BlaC based on the wt structure). Orange, CSP of >0.2 ppm; yellow, 0.2 ppm ≥ CSP > 0.05 ppm; blue, CSP of ≤0.05 ppm; red, peak broadened beyond detection in G132N but not in wt BlaC; black, no data available. Side chains of the mutated residues are displayed in a magenta stick representation. Ser70 is shown in cyan sticks.

The positions of the backbone amide resonances in the spectra of both mutants were compared with those of wt BlaC (Fig. 5). Large and extensive chemical shift perturbations (CSPs) were observed for both mutants, which we attribute to significant disturbances of the extended active-site hydrogen-bonding network involving side chains, amides, carbonyls, and water molecules. No large perturbations were observed for any residues that are very far from the mutation site, further corroborating that the proteins are correctly folded.

In the NMR spectrum of wt BlaC, the resonances of the amides of 4 residues close to the phosphate-binding site are missing. The K234R mutation is near the phosphate-binding site (26) and appears to affect the dynamic behavior of at least Thr235, which participates in phosphate binding. To determine whether the affinity and kinetics of phosphate binding were affected by the mutation, phosphate titration was carried out. An equilibrium dissociation constant (*K_D_*) of 20.1 ± 0.7 mM (Fig. S6) (error from the fit of one titration) was found for BlaC K234R, which is close to the *K_D_* of 27 ± 11 mM (error from duplicate titrations) found for wt BlaC (26). Analysis using TITAN software (27) showed that the off-rate of the phosphate from the BlaC K234R-binding site is very high, >2 × 10^4^ s^−1^, as it is for wt BlaC (Fig. S7). Such fast exchange cannot explain the broadening beyond the detection of the resonances in the phosphate-binding site. Another process must be the reason why these resonances are absent in the spectra of both wt and K234R BlaC.

To probe the effect of the mutations on the pico- to nanosecond dynamics, the nuclear Overhauser effect (NOE) of the backbone amides was measured (Fig. S8). Most NOEs are close to 1, indicating that the two mutants, like wt BlaC, are overall very rigid on this time scale. The NOEs of most residues are similar to those of the wt, showing the expected reductions for flexible loops and high values in long β-sheets and α-helices. There are also residues that show increased or decreased rigidity. Notably, residue Ile247 in BlaC variant K234R displays a very low NOE. This amide is situated on β-strand 4, directly adjacent to residue 234 on β-strand 3. In the wt structure, the Ile247 amide is hydrogen bonded to the carbonyl group of Lys234. The loss of rigidity that we observe may suggest that this hydrogen bond is lost upon mutation of Lys234 to Arg. However, the other amides on these two strands show NOEs that are similar to those in wt BlaC, indicating that the effect is very local. In G132N, fast dynamics are observed around Ser70, at least for one of the two forms. Val80, which displays increased flexibility in wt BlaC, is stabilized in G132N and even further in K234R, where it returns to a normal rigidity compared to the rest of the protein.

**FIG 6:**
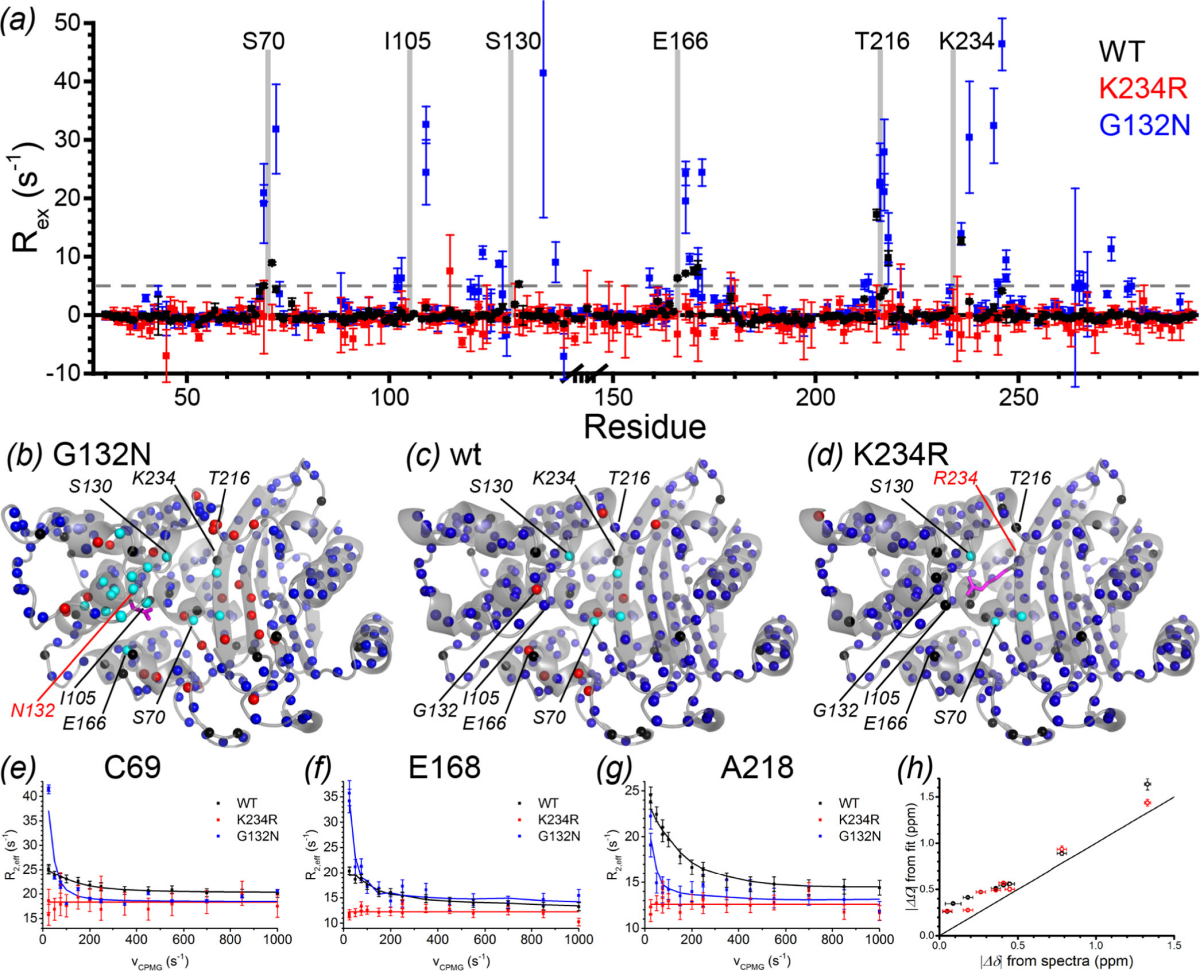
Chemical exchange effects observed for backbone ^15^N resonances in BlaC, as measured by CPMG relaxation dispersion analysis. *R_ex_* is defined as the *R*_2,_*_eff_* at ν_CPMG_ = 25 s^−1^ minus that at 1,000 s^−1^. (a) Plot of *R_ex_* at 20.0 T versus the residue number. The break on the horizontal axis represents the BlaC-specific G-G-G-T loop, which is not present in the Ambler numbering. Error bars represent the 95% confidence intervals based on three duplicate delays per experiment. (b to d) Color map of *R_ex_* on the structure of BlaC G132N (PBD accession number 7A74 [this work]) (b) and on chain A (PBD accession number 5NJ2 [26]) for wt BlaC (c) and the model of K234R BlaC (d). Backbone amides with an *R_ex_* of >5 s^−1^ are displayed in red, and those with an *R_ex_* of ≤5 s^−1^ are in blue. Amides for which the resonance was broadened beyond detection are displayed in cyan. Amides for which no data were available for a different reason (e.g., proline or too much overlap) are displayed in black. Mutated residues are displayed as magenta sticks. (e to g) Examples of CPMG profiles from BlaC wt, G132N, and K234R measured at 20.0 T. The lines represent grouped two-field fits for relaxation dispersion for BlaC wt and G132N data and linear fits for BlaC K234R. Error bars represent standard deviations based on three duplicate frequencies. (h) Correlation plot of the ^15^N |ΔΩ| derived from two-field relaxation dispersion NMR fits of the separate state A (black) and state B (red) versus the ^15^N chemical shift differences observed in the ^1^H-^15^N TROSY-HSQC spectrum for the two BlaC G132N states. The diagonal line indicates *y* = *x*. Vertical and horizontal error bars represent standard deviations of the fit and estimated errors in peak picking, respectively.

To probe the effect of the G132N and K234R mutations on the millisecond dynamics of BlaC, Carr-Purcell-Meiboom-Gill (CPMG) relaxation dispersion experiments were performed. In resting-state wt BlaC, significant relaxation dispersion effects were observed around the active site, indicating dynamics with an exchange rate of ca. 860 s^−1^ (Fig. 6a, black) (10). Surprisingly, this relaxation dispersion is completely absent in mutant K234R (Fig. 6a, red). The opposite effect is observed in mutant G132N, which shows strikingly increased relaxation dispersion for many residues (Fig. 6a, blue). Like the broadened and split peaks, the peaks that show relaxation dispersion are also centered around the active site (Fig. 6b). Comparisons of CPMG profiles for wt BlaC and the two mutants are shown for three residues in Fig. 6e to g. The data acquired for BlaC G132N at 20.0 T (850 MHz) and 22.3 T (950 MHz) were consistent. Example curves are shown in Fig. S9.

**FIG 7:**
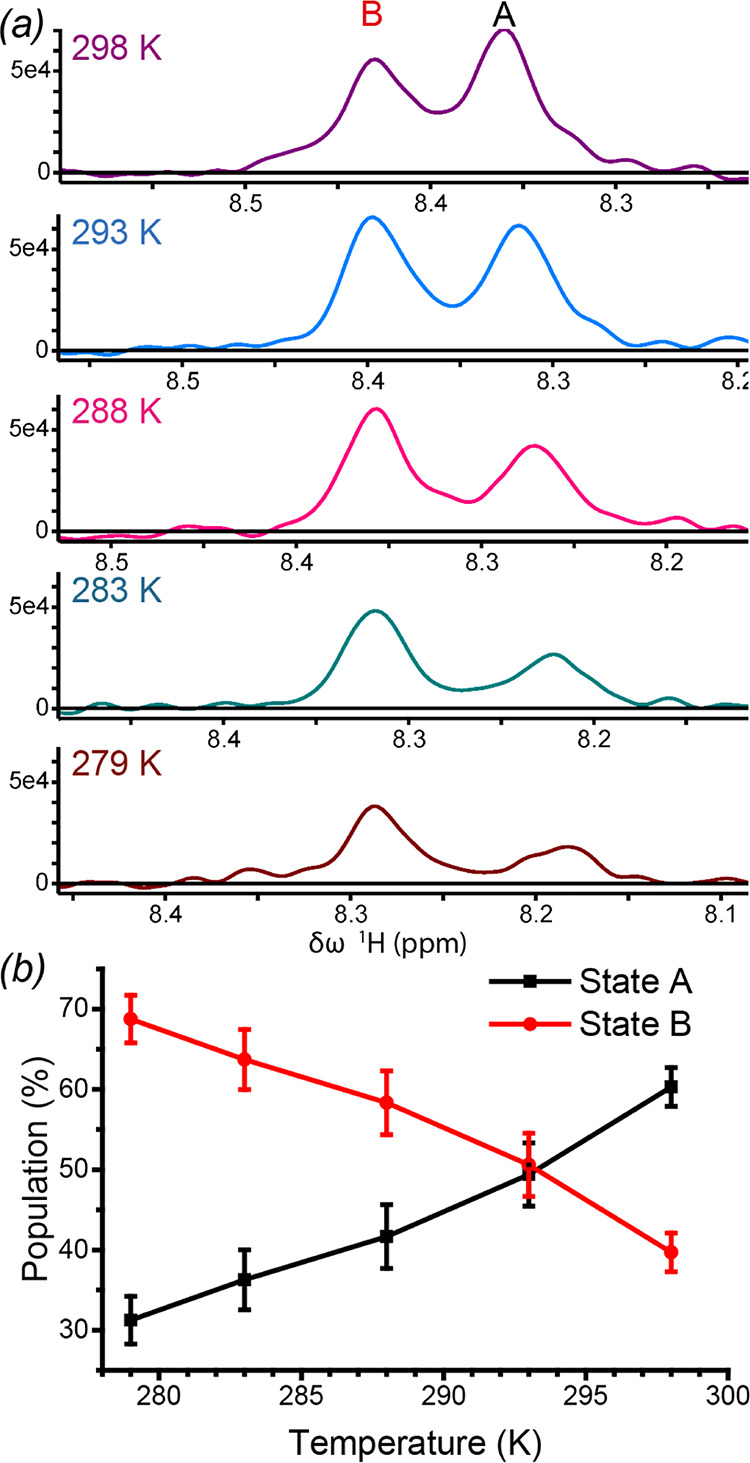
Temperature dependence of the BlaC G132N state B population. (a) Example of a doubled peak, Ala164, at five temperatures. The |ΔΩ| in the ^15^N dimension is zero for this amide, as is the ^15^N Δδ in the temperature titration; thus, slices through this dimension yield the peak shapes and maxima for both states A and B. Note that peak maxima decrease with temperature as a result of increasing sample viscosity. (b) Averages and standard deviations of the relative intensities of 10 split-backbone amide resonances in the five ^1^H-^15^N TROSY-HSQC spectra.

In the BlaC G132N spectrum, two peaks are visible for many of the residues that show relaxation dispersion. Thus, if these peaks were used together in a grouped fit, not all peaks represent state A because some will be from state B, and therefore, the globally fitted population of the minor state, *p_B_*, will be wrong. To distinguish which peaks belong to which state, transverse relaxation optimized spectroscopy-heteronuclear single quantum coherence (TROSY-HSQC) spectra were recorded at five temperatures ranging from 279 to 298 K. The relative intensities of all doubled peaks were found to be affected equally, indicating that they all correspond to two states of the same chemical exchange phenomenon. The relative intensities of 10 nonoverlapping peak pairs were used to determine that the population of state B at 298 K is 40% ± 2%. Furthermore, this analysis revealed that the major state at 298 K becomes the minor state at lower temperatures (Fig. 7). Analysis of the relaxation dispersion results (Table S3 and Fig. S9) yields an exchange rate of 88 ± 6 s^−1^, corresponding with our estimate of <100 s^−1^ based on the relative positions of split peaks in the TROSY-HSQC spectra. The relaxation dispersion analysis also yields an estimate of the absolute ^15^N chemical shift difference, |ΔΩ|, between the resonance positions of the two states. The fitted |ΔΩ| values correlate well with the chemical shift differences between peaks as observed in the TROSY-HSQC spectra (Fig. 6h), although there appears to be a small bias, reflecting either an experimental underestimation of |Δδ| due to peak-picking artifacts of partly overlapping peaks or an overestimation of the |ΔΩ| by the fitting procedure. The results clearly suggest that the observed peak doubling reflects the same exchange process that was observed with the relaxation dispersion experiments.

Chemical exchange with a rate of ca. 88 s^−1^ should also be observable using the chemical exchange saturation transfer (CEST) method. We previously reported no measurable exchange in this regime for wt BlaC (10). CEST of the G132N mutant was measured, yielding direct observations of exchange between the resonances with the largest chemical shift differences, Gly238 and Asp246 (Fig. S10). Unfortunately, the |ΔΩ| values of the other exchanging resonances are too low to be observed clearly via CEST, so no other CEST profiles indicated exchange. Nevertheless, it is clear that the doubled peaks in the G132N spectrum arise from two states of the protein that are in exchange. The phenomenon of split resonance peaks in the G132N spectra is reminiscent of that in the clavulanic acid adduct-bound wt protein (10). However, the relative positions of the peaks are not similar, nor could any exchange be determined between the adduct-bound states. Moreover, the number of observed states for the adduct-bound protein is not the same as that for the G132N mutant. It therefore seems unlikely that these observations represent the same dynamic process.

## DISCUSSION

Here, we report on the dynamics of two variants of BlaC, each of which increases resistance to inhibition by clavulanic acid, and a high-resolution crystal structure of one of them. In the laboratory evolution experiment aimed at finding such variants, we found only one mutant that showed considerably less inhibition by clavulanic acid. In our experience, we usually find more mutants when screening for increased activity against specific β-lactam antibiotics. Also, some other variants that reduce clavulanic acid inhibition have been reported for other β-lactamases, S130G (28), T237A (29), C69L (20), and R220S (20). However, clavulanic acid is a small molecule, and the secondary chemistry that occurs upon acylation is the main reason for its inhibitory effect. It could be that few variants are possible that either reduce binding or enhance the hydrolysis of the adduct.

Curiously, the mutations G132N and K234R have opposite effects on the dynamic behavior in the millisecond time scale. K234R reduces the active-site chemical exchange in the millisecond time scale, while G132N increases it. Neither mutation has large effects on the pico- to nanosecond time-scale dynamics of BlaC. The NMR data show that BlaC G132N exists in two almost equally populated states, exchanging with a rate of ca. 88 s^−1^ at 298 K (sum of the forward and backward rates). This is observed through the splitting of backbone amide resonance peaks around the active site and the mutation as well as through relaxation dispersion experiments. Additionally, the resonances of amides in a large region around the mutation are broadened beyond detection.

The crystal structure also suggests an increase in flexibility because several residues occur in multiple conformers. The relative occupancies of the Ser104 and Ser130 conformations observed in the crystal structure match those of the states observed in the NMR experiments. Moreover, these residues are positioned right in the region of the protein where broadening and exchange were observed with NMR. The X-ray and NMR observations may thus represent the same phenomenon. In this case, the two conformations, which are slowly exchanging at room temperature, were captured by flash freezing of the crystal prior to X-ray analysis. The region in which the effects of conformational exchange are observed with NMR is relatively large for single side chain movements, and the exchange is relatively slow. These observations suggest the possibility that the double conformations captured in the crystal may represent the observable part of a mixed conformational and electrostatic exchange affecting the entire extended hydrogen-bonding network of the active site and α-domain.

The question remains if the exchange process is involved in the functional differences between the enzyme and the mutant, i.e., increased clavulanate resistance and avibactam affinity, or if it is an unrelated side effect of the mutation. The two Ser130 orientations observed in the G132N structure are reminiscent of observations in similarly clavulanic acid-resistant variants of SHV, PSE-4, and CTX-M-14 β-lactamases. There, molecular dynamics (MD) simulations (19) and X-ray crystallography (18, 30, 31) have shown that the presence of Arg234 rather than Lys234 causes a displacement of the Ser130 side chain, moving it further away from the reactive Ser70. This may prevent cross-linking between Ser70 and Ser130, which could explain the increased resistance of the enzyme to clavulanic acid. In this light, our results suggest that the same mechanism might apply for BlaC and could explain the resistance of both the G132N and K234R mutants. In principle, the subtle change in the population of the two states as a function of temperature (Fig. 7) could thus be reflected in different degrees of sensitivity for clavulanic acid inhibition, but such experiments are complicated by the general effects of temperature on the conversion rates.

The dynamic behavior of wt BlaC on the millisecond time scale (10) is not detectable in mutant K234R. This finding indicates that millisecond chemical exchange is not required for clavulanate hydrolysis. Moreover, as this mutant is able to hydrolyze at least ampicillin, benzylpenicillin, cephalothin (20), and nitrocefin, it presents direct evidence that the millisecond dynamics are not required for β-lactamase activity. Doucet et al. first reported that changes in millisecond dynamics in TEM-1 correlate with functional differences (32) but later observed that laboratory-engineered chimeric β-lactamases displayed conserved function but strikingly increased dynamic behavior (33, 34). To our knowledge, BlaC K234R is the first functional class A β-lactamase in which no millisecond dynamics could be detected in or near the active site. It is noted that dynamics that are much faster (middle- to low-microsecond time scale) could be present because motions in this time range are not picked up with the NMR methods applied in this study. Still, our findings place BlaC, and perhaps all β-lactamases, in the group of enzymes for which millisecond dynamics do not play a role in catalysis. In enzymes with multistep reactions, involving more than a single substrate or with open and closed states, motions are often essential for the enzyme to proceed from one step of the reaction to the next. Well-studied examples comprise adenylate kinase, dihydrofolate reductase, and cytochrome P450cam (35–42). Enzymes that catalyze relatively simple reactions and have an exposed active site may not have taken advantage of the dynamics naturally present in protein molecules to enhance their function (43). This is of importance for drug design because the development of inhibitors or new β-lactam antibiotics can be based on the crystal structures of β-lactamases only, and potential lowly populated states with different conformations are unlikely to be relevant for function. However, dynamics can still be an important property for evolvability. Conformations that occur with a low population can have low-level catalytic activity on other substrates, forming the raw material of evolution to develop enzymes with different specificities. Evolving a completely rigid enzyme could therefore be a dead-end street, even if the current function requires no dynamics (44, 45).

## MATERIALS AND METHODS

### Error-prone PCR and functional screening.

Error-prone PCR mutagenesis was performed on an Escherichia coli expression-optimized DNA sequence (see Fig. S1 in the supplemental material) encoding the soluble domain of BlaC, using DreamTaq polymerase (0.1 U/μl; Thermo Fisher Scientific) with the reaction conditions recommended by the supplier but disturbed by the addition of manganese (0.2 mM), extra magnesium (2 mM), and skewed nucleotide concentrations (0.52 mM dCTP/dTTP versus 0.2 mM dATP/dGTP) in the reaction mixture. The primers that were used are listed in Table S1. Fifteen rounds of error-prone PCR were followed by purification of the reaction product using the Illustra GFX PCR DNA and gel band purification kit (GE Lifesciences) and another 25 rounds of non-error-prone PCR to further amplify the product. Primers for the second PCR (Table S1) anneal only to the product of the first reaction, not to the template. The product of the second PCR was purified as described above and subsequently digested using the restriction enzymes BglII and XhoI, with the addition of DpnI to digest any original template. The resulting gene fragments, containing only the coding sequence of soluble BlaC with semirandom combinations of mutations, were purified by agarose gel band extraction. These were subsequently cloned into similarly digested and purified pUK21-based vectors containing expression and subcellular localization elements to create a cloning/expression plasmid capable of isopropyl-β-d-thiogalactopyranoside (IPTG)-induced expression of BlaC mutants fused to an N-terminal, TorA-derived protein export signal for use in E. coli. A map of the construct is shown in Fig. S2. These plasmids were introduced into E. coli strain KA797 (46) using the chemical transformation method described previously by Inoue et al. (47), yielding libraries of up to 0.5 million CFU per 200 μl of transformation solution. Screening for clavulanate resistance was performed by incubation at 37°C overnight on LB agar containing 50 μg/ml kanamycin sulfate, 1 mM IPTG, 8 μg/ml ampicillin, and 1 μg/ml clavulanic acid. Colonies were transferred to separate liquid cultures, and after overnight incubation, plasmids were isolated from the cells and used to transform fresh cells. Hits were registered as valid only if the plasmid also transmitted the same ampicillin/clavulanic acid-resistant phenotype to the fresh cells. The mutation frequency and bias in the library were controlled by Sanger sequencing of at least 10 randomly picked colonies from plates without ampicillin and clavulanic acid. The library harbored on average 5 single nucleotide replacements and ∼0.08 deletions per mutant (Fig. S3a). The mutations were found to be heavily biased toward A>G/T>C and A>T/T>A mutations and against G>C/C>G, G>T/C>A, and A>C/T>G mutations, with only G>A/C>T mutations having an average frequency (Fig. S3b). This is in line with expectations, based on observations by others (e.g., see references 48 and 49). The efficiency of the transformation was identified to be the bottleneck in library generation. Using E. coli strain KA797 and the transformation protocol described previously by Inoue et al. (47), efficiencies of up to 0.5 million CFU per 200 μl of cell suspension were obtained. More details are provided in Text S1 in the supplemental material.

### Site-directed mutagenesis.

Site-directed mutations in the *blaC* gene were made using a whole-plasmid synthesis approach, with the primers listed in Table S1. The incorporation of the correct mutations and the absence of any other mutations were checked by comparison of Sanger sequencing data of each mutant from two sides (Baseclear BV, Leiden, The Netherlands).

### Protein production and purification.

Pure wt and variant BlaC proteins, without a signal peptide and without a purification tag (residues 43 to 407 of the structure under UniProt accession number P9WKD3), but with an N-terminal Gly residue, were obtained as described previously (26). In short, the proteins fused to an N-terminal 6×His purification tag were produced in E. coli and purified using nickel affinity chromatography, and the purification tag was then cleaved off, followed by inverse nickel affinity chromatography to yield the pure protein. The Ambler standard β-lactamase numbering scheme (50) is used throughout the text.

### Crystallization.

Crystallization conditions for the BlaC G132N and K234R mutants were screened by sitting-drop vapor diffusion using the BCS, Morpheus, JCSG+, and PACT premier (Molecular Dimensions) screens at 293 K with 100-nl drops at a 1:1 ratio (51). The plates were pipetted by using the NT8 drop setter (Formulatrix). BlaC K234R was used at concentrations of 8 mg ml^−1^ and 10 mg ml^−1^ in 100 mM morpholineethanesulfonic acid (MES)-NaOH buffer (pH 6.4). Multiple conditions across the screens showed growth on microneedles or multilayer plates, which were used for seeding and further buffer optimization. However, that did not yield any usable crystals. BlaC G132N was used at a concentration of 18 mg ml^−1^ in 100 mM MES-NaOH buffer (pH 6.4). To obtain crystals, it was necessary to supplement the protein with 100 mM sodium phosphate buffer and cross-seed with crystals from another BlaC mutant. A crystal of BlaC G132N grew within 2 months in 0.1 M Morpheus buffer 1 (pH 6.5) with 0.09 M halogens and 30% (wt/vol) ethylene glycol polyethylene glycol 8000 as the precipitant. The crystal was mounted on a cryoloop in mother liquor with additional 20% glycerol and vitrified in liquid nitrogen for data collection.

### X-ray data collection, processing, and structure solution.

X-ray diffraction data were obtained from a single crystal at the Diamond Light Source (DLS), Oxford, United Kingdom. The crystallographic diffraction data were recorded on a Pilatus detector to a resolution of 1.18 Å. Data were processed and integrated with XDS (52) and scaled with AIMLESS (53); the resolution was set to 1.55 Å based on |*I*/σ*I*| and CC_1/2_ values. The structure was solved by molecular replacement using MOLREP (54) from the CCP4 suite (54) using the structure under PDB accession number 2GDN (11) as a search model. Subsequently, building and refinement were performed using Coot and REFMAC (54). Multiple residues were modeled in double conformations, namely, K93, D100, S104, S130, V263, and M264. Residues N214-T215-T216 exist in multiple conformations and were modeled in two representative conformations. The model was further optimized using the PDB-REDO Web server (55). Structure validation showed a Ramachandran Z-score (56) of −0.643; 98% of all residues are within the Ramachandran plot-favored regions with 2 outliers, and according to MolProbity (57), the structure belongs to the 100th percentile. Data collection and refinement statistics can be found in Table S2.

### NMR spectroscopy.

Unless mentioned otherwise, all NMR spectra were recorded on a Bruker AVIII HD 850-MHz (20.0-T) spectrometer equipped with a TCI cryoprobe, and all experiments were performed on samples containing 0.38 mM ^15^N-enriched BlaC in a solution containing 94 mM sodium MES (pH 6.4) and 6% D_2_O at 298 K. HNCa spectra were measured on samples containing 0.6 and 0.28 mM ^15^N,^13^C-enriched BlaC G132N and K234R, respectively, in the same buffer. These spectra were recorded using the standard Bruker pulse program “trhncaetgp3d,” processed with Topspin 3.2 (BioSpin; Bruker, Leiderdorp, The Netherlands), and analyzed using CCPNmr analysis (58). Resonance assignment was performed by comparison of the HNCa to the assigned wt spectra (Biological Magnetic Resonance Data Bank [BMRB] accession number 27888 [https://bmrb.io/data_library/summary/index.php?bmrbId=27888]) (26). NOE measurements were performed using the standard Bruker pulse program “hsqcnoef3gpsi” with a ^1^H saturation delay of 4 s. NOE data were processed with Topspin 3.2, and the resulting peak heights were fitted to exponential decay curves using Dynamics Center 2.5 (BioSpin; Bruker, Rheinstetten, Germany).

Carr-Purcell-Meiboom-Gill (CPMG) relaxation dispersion measurements were performed using the TROSY CPMG pulse program as detailed previously by Vallurupalli et al. (59), with two blocks of 0, 1 (2×), 2, 3 (2×), 4, 6, 8, 10 (2×), 14, 18, 22, 28, 34, and 40 ^15^N 180° pulses in a 40-ms relaxation time. For mutant G132N, an additional CPMG relaxation dispersion experiment was performed at a second magnetic field, using a Bruker AVIII HD 950-MHz (22.3-T) spectrometer equipped with a TCI cryoprobe and a 0.54 mM protein sample. Data were processed with NMRPipe (60), and the resulting resonances were fitted to a glore line shape using FuDa (61). Effective transverse relaxation rates (*R*_2,_*_eff_*) were calculated from the fitted peak heights using the formula *R*_2,_*_eff_*(ν_CPMG_) = −ln[*I*(ν_CPMG_)/*I*_0_]/*T_ex_*. Rate and absolute ^15^N chemical shift differences of the chemical exchange were determined using two-field grouped fits to the Bloch-McConnell equations with CATIA software (61). Two separate grouped fits were performed for states “A” and “B” in the G132N spectra, with the second-state population *p_B_* fixed at 0.4 and 0.6, respectively. Chemical exchange saturation transfer (CEST) measurements were performed using the standard Bruker “hsqc_cest_etf3gpsitc3d” pulse program, with a 2.5-s recycle delay, 0.8-s *B*_1_ irradiation at all frequencies in the ^15^N range of 100.5:0.5:130 ppm, and field powers of 8 Hz and 26 Hz.

Figures containing protein structures were created using the PyMOL molecular graphics system, version 2.2 (Schrödinger, LLC).

### Data availability.

NMR chemical shift assignments and relaxation data have been submitted to the Biological Magnetic Resonance Data Bank (BMRB) and can be accessed under BMRB accession numbers 27889 (G132N) (https://bmrb.io/data_library/summary/index.php?bmrbId=27889) and 27891 (K234R) (https://bmrb.io/data_library/summary/index.php?bmrbId=27891). The BlaC G132N crystal structure and data files have been submitted to the Protein Data Bank (PDB) and can be accessed under accession number 7A74.

## References

[1] Frick IM, Wikström M, Forsén S, Drakenberg T, Gomi H, Sjöbring U, Björck L. 1992. Convergent evolution among immunoglobulin G-binding bacterial proteins. Proc Natl Acad Sci U S A 89:8532–8536. 10.1073/pnas.89.18.8532. 1528858 PMC49954

[2] Sikosek T, Krobath H, Chan HS. 2016. Theoretical insights into the biophysics of protein bi-stability and evolutionary switches. PLoS Comput Biol 12:e1004960. 10.1371/journal.pcbi.1004960. 27253392 PMC4890782

[3] Soroka D, Ourghanlian C, Compain F, Fichini M, Dubée V, Mainardi J-L, Hugonnet J-E, Arthur M. 2017. Inhibition of β-lactamases of mycobacteria by avibactam and clavulanate. J Antimicrob Chemother 72:1081–1088. 10.1093/jac/dkw546. 28039278

[4] Tokuriki N, Tawfik DS. 2009. Protein dynamism and evolvability. Science 324:203–207. 10.1126/science.1169375. 19359577

[5] Zou T, Risso VA, Gavira JA, Sanchez-Ruiz JM, Ozkan SB. 2015. Evolution of conformational dynamics determines the conversion of a promiscuous generalist into a specialist enzyme. Mol Biol Evol 32:132–143. 10.1093/molbev/msu281. 25312912

[6] Campbell E, Kaltenbach M, Correy GJ, Carr PD, Porebski BT, Livingstone EK, Afriat-Jurnou L, Buckle AM, Weik M, Hollfelder F, Tokuriki N, Jackson CJ. 2016. The role of protein dynamics in the evolution of new enzyme function. Nat Chem Biol 12:944–950. 10.1038/nchembio.2175. 27618189

[7] González MM, Abriata LA, Tomatis PE, Vila AJ. 2016. Optimization of conformational dynamics in an epistatic evolutionary trajectory. Mol Biol Evol 33:1768–1776. 10.1093/molbev/msw052. 26983555 PMC5854100

[8] Petrović D, Frank D, Kamerlin SCL, Hoffmann K, Strodel B. 2017. Shuffling active site substate populations affects catalytic activity: the case of glucose oxidase. ACS Catal 7:6188–6197. 10.1021/acscatal.7b01575. 29291138 PMC5745072

[9] Petrović D, Risso VA, Kamerlin SCL, Sanchez-Ruiz JM. 2018. Conformational dynamics and enzyme evolution. J R Soc Interface 15:20180330. 10.1098/rsif.2018.0330. 30021929 PMC6073641

[10] Elings W, Gaur A, Blok AJ, Timmer M, van Ingen H, Ubbink M. 2020. β-Lactamase of Mycobacterium tuberculosis shows dynamics in the active site that increase upon inhibitor binding. Antimicrob Agents Chemother 64:e02025-19. 10.1128/AAC.02025-19. 31871087 PMC7038250

[11] Wang F, Cassidy C, Sacchettini JC. 2006. Crystal structure and activity studies of the *Mycobacterium tuberculosis* β-lactamase reveal its critical role in resistance to β-lactam antibiotics. Antimicrob Agents Chemother 50:2762–2771. 10.1128/AAC.00320-06. 16870770 PMC1538687

[12] Soroka D, De La Sierra-Gallay IL, Dubée V, Triboulet S, Van Tilbeurgh H, Compain F, Ballell L, Barros D, Mainardi JL, Hugonnet JE, Arthur M. 2015. Hydrolysis of clavulanate by *Mycobacterium tuberculosis* β-lactamase BlaC harboring a canonical SDN motif. Antimicrob Agents Chemother 59:5714–5720. 10.1128/AAC.00598-15. 26149997 PMC4538473

[13] Ourghanlian C, Soroka D, Arthur M. 2017. Inhibition by avibactam and clavulanate of the β-lactamases KPC-2 and CTX-M-15 harboring the substitution N^132^G in the conserved SDN motif. Antimicrob Agents Chemother 61:e02510-16. 10.1128/AAC.02510-16. 28069651 PMC5328567

[14] Flores AR, Parsons LM, Pavelka MS. 2005. Genetic analysis of the beta-lactamases of *Mycobacterium tuberculosis* and *Mycobacterium smegmatis* and susceptibility to beta-lactam antibiotics. Microbiology 151:521–532. 10.1099/mic.0.27629-0. 15699201

[15] Galleni M, Franceschini N, Quinting B, Fattorini L, Orefici G, Oratore A, Frère JM, Amicosante G. 1994. Use of the chromosomal class A beta-lactamase of Mycobacterium fortuitum D316 to study potentially poor substrates and inhibitory beta-lactam compounds. Antimicrob Agents Chemother 38:1608–1614. 10.1128/AAC.38.7.1608. 7979294 PMC284600

[16] Sauvage E, Fonzé E, Quinting B, Galleni M, Frère J-M, Charlier P. 2006. Crystal structure of the Mycobacterium fortuitum class A β-lactamase: structural basis for broad substrate specificity. Antimicrob Agents Chemother 50:2516–2521. 10.1128/AAC.01226-05. 16801434 PMC1489783

[17] Lenfant F, Labia R, Masson JM. 1991. Replacement of lysine 234 affects transition state stabilization in the active site of beta-lactamase TEM1. J Biol Chem 169:17187–17194. 1910040

[18] Winkler ML, Rodkey EA, Taracila MA, Drawz SM, Bethel CR, Papp-Wallace KM, Smith KM, Xu Y, Dwulit-Smith JR, Romagnoli C, Caselli E, Prati F, van den Akker F, Bonomo RA. 2013. Design and exploration of novel boronic acid inhibitors reveals important interactions with a clavulanic acid-resistant sulfhydryl-variable (SHV) β-lactamase. J Med Chem 56:1084–1097. 10.1021/jm301490d. 23252553 PMC3943433

[19] Mendonça N, Manageiro V, Robin F, Salgado MJ, Ferreira E, Caniça M, Bonnet R. 2008. The Lys234Arg substitution in the enzyme SHV-72 is a determinant for resistance to clavulanic acid inhibition. Antimicrob Agents Chemother 52:1806–1811. 10.1128/AAC.01381-07. 18316518 PMC2346665

[20] Egesborg P, Carlettini H, Volpato JP, Doucet N. 2015. Combinatorial active-site variants confer sustained clavulanate resistance in BlaC β-lactamase from *Mycobacterium tuberculosis*. Protein Sci 24:534–544. 10.1002/pro.2617. 25492589 PMC4380984

[21] Papp-Wallace KM, Winkler ML, Taracila MA, Bonomo RA. 2015. Variants of β-lactamase KPC-2 that are resistant to inhibition by avibactam. Antimicrob Agents Chemother 59:3710–3717. 10.1128/AAC.04406-14. 25666153 PMC4468660

[22] Feiler C, Fisher AC, Boock JT, Marrichi MJ, Wright L, Schmidpeter PAM, Blankenfeldt W, Pavelka M, DeLisa MP. 2013. Directed evolution of *Mycobacterium tuberculosis* β-lactamase reveals gatekeeper residue that regulates antibiotic resistance and catalytic efficiency. PLoS One 8:e73123. 10.1371/journal.pone.0073123. 24023821 PMC3762836

[23] Jelsch C, Mourey L, Masson J-M, Samama J-P. 1993. Crystal structure of *Escherichia coli* TEM1 β-lactamase at 1.8 Å resolution. Proteins 16:364–383. 10.1002/prot.340160406. 8356032

[24] Kuzin AP, Nukaga M, Nukaga Y, Hujer AM, Bonomo RA, Knox JR. 1999. Structure of the SHV-1 β-lactamase. Biochemistry 38:5720–5727. 10.1021/bi990136d. 10231522

[25] van Beusekom LMJ. 2019. Improving protein structure with homology-based information and prior knowledge. Doctorate thesis. Utrecht University, Utrecht, The Netherlands.

[26] Elings W, Tassoni R, Van Der Schoot SA, Luu W, Kynast JP, Dai L, Blok AJ, Timmer M, Florea BI, Pannu NS, Ubbink M. 2017. Phosphate promotes the recovery of Mycobacterium tuberculosis β-lactamase from clavulanic acid inhibition. Biochemistry 56:6257–6267. 10.1021/acs.biochem.7b00556. 29087696 PMC5707625

[27] Waudby CA, Ramos A, Cabrita LD, Christodoulou J. 2016. Two-dimensional NMR lineshape analysis. Sci Rep 6:24826. 10.1038/srep24826. 27109776 PMC4843008

[28] Sirot D, Labia R, Pouedras P, Chanal-Claris C, Cerceau C, Sirot J. 1998. Inhibitor-resistant OXY-2-derived β-lactamase produced by Klebsiella oxytoca. Antimicrob Agents Chemother 42:2184–2187. 10.1128/AAC.42.9.2184. 9736532 PMC105771

[29] Kurz SG, Wolff KA, Hazra S, Bethel CR, Hujer AM, Smith KM, Xu Y, Tremblay LW, Blanchard JS, Nguyen L, Bonomo RA. 2013. Can inhibitor-resistant substitutions in the Mycobacterium tuberculosis β-lactamase BlaC lead to clavulanate resistance? A biochemical rationale for the use of β-lactam–β-lactamase inhibitor combinations. Antimicrob Agents Chemother 57:6085–6096. 10.1128/AAC.01253-13. 24060876 PMC3837893

[30] Lim D, Sanschagrin F, Passmore L, De Castro L, Levesque RC, Strynadka NCJ. 2001. Insights into the molecular basis for the carbenicillinase activity of PSE-4 β-lactamase from crystallographic and kinetic studies. Biochemistry 40:395–402. 10.1021/bi001653v. 11148033

[31] Soeung V, Lu S, Hu L, Judge A, Sankaran B, Prasad BVV, Palzkill T. 2020. A drug-resistant β-lactamase variant changes the conformation of its active site proton shuttle to alter substrate specificity and inhibitor potency. J Biol Chem 295:18239–18255. 10.1074/jbc.RA120.016103.33109613

[32] Doucet N, Savard P-Y, Pelletier JN, Gagné SM. 2007. NMR investigation of Tyr105 mutants in TEM-1 beta-lactamase: dynamics are correlated with function. J Biol Chem 282:21448–21459. 10.1074/jbc.M609777200. 17426035

[33] Gobeil SMC, Clouthier CM, Park J, Gagné D, Berghuis AM, Doucet N, Pelletier JN. 2014. Maintenance of native-like protein dynamics may not be required for engineering functional proteins. Chem Biol 21:1330–1340. 10.1016/j.chembiol.2014.07.016. 25200606

[34] Gobeil SMC, Ebert MCCJC, Park J, Gagné D, Doucet N, Berghuis AM, Pleiss J, Pelletier JN. 2019. The structural dynamics of engineered β-lactamases vary broadly on three timescales yet sustain native function. Sci Rep 9:6656. 10.1038/s41598-019-42866-8. 31040324 PMC6491436

[35] Henzler-Wildman KA, Thai V, Lei M, Ott M, Wolf-Watz M, Fenn T, Pozharski E, Wilson MA, Petsko GA, Karplus M, Hübner CG, Kern D. 2007. Intrinsic motions along an enzymatic reaction trajectory. Nature 450:838–844. 10.1038/nature06410. 18026086

[36] Wolf-Watz M, Thai V, Henzler-Wildman K, Hadjipavlou G, Eisenmesser EZ, Kern D. 2004. Linkage between dynamics and catalysis in a thermophilic-mesophilic enzyme pair. Nat Struct Mol Biol 11:945–949. 10.1038/nsmb821. 15334070

[37] Saavedra HG, Wrabl JO, Anderson JA, Li J, Hilser VJ. 2018. Dynamic allostery can drive cold adaptation in enzymes. Nature 558:324–328. 10.1038/s41586-018-0183-2. 29875414 PMC6033628

[38] Boehr DD, McElheny D, Dyson HJ, Wright PE. 2006. The dynamic energy landscape of dihydrofolate reductase catalysis. Science 313:1638–1642. 10.1126/science.1130258. 16973882

[39] Boehr DD, McElheny D, Dyson HJ, Wright PE. 2010. Millisecond timescale fluctuations in dihydrofolate reductase are exquisitely sensitive to the bound ligands. Proc Natl Acad Sci U S A 107:1373–1378. 10.1073/pnas.0914163107. 20080605 PMC2824364

[40] Oyen D, Fenwick RB, Aoto PC, Stanfield RL, Wilson IA, Dyson HJ, Wright PE. 2017. Defining the structural basis for allosteric product release from E. coli dihydrofolate reductase using NMR relaxation dispersion. J Am Chem Soc 139:11233–11240. 10.1021/jacs.7b05958. 28737940 PMC5562155

[41] Follmer AH, Mahomed M, Goodin DB, Poulos TL. 2018. Substrate-dependent allosteric regulation in cytochrome P450cam (CYP101A1). J Am Chem Soc 140:16222–16228. 10.1021/jacs.8b09441. 30376314

[42] Follmer AH, Tripathi S, Poulos TL. 2019. Ligand and redox partner binding generates a new conformational state in cytochrome P450cam (CYP101A1). J Am Chem Soc 141:2678–2683. 10.1021/jacs.8b13079. 30672701 PMC8917899

[43] Ben Bdira F, Waudby CA, Volkov AN, Schröder SP, Ab E, Codée JDC, Overkleeft HS, Aerts JMFG, van Ingen H, Ubbink M. 2020. Dynamics of ligand binding to a rigid glycosidase. Angew Chem Int Ed Engl 59:20508–20514. 10.1002/anie.202003236. 32533782 PMC7693232

[44] Klinman JP, Kohen A. 2014. Evolutionary aspects of enzyme dynamics. J Biol Chem 289:30205–30212. 10.1074/jbc.R114.565515. 25210031 PMC4215204

[45] Pabis A, Risso VA, Sanchez-Ruiz JM, Kamerlin SC. 2018. Cooperativity and flexibility in enzyme evolution. Curr Opin Struct Biol 48:83–92. 10.1016/j.sbi.2017.10.020. 29141202

[46] Coulondre C, Miller JH. 1977. Genetic studies of the *lac* repressor. IV. Mutagenic specificity in the *lacI* gene of *Escherichia coli*. J Mol Biol 117:577–606. 10.1016/0022-2836(77)90059-6. 416218

[47] Inoue H, Nojima H, Okayama H. 1990. High efficiency transformation of *Escherichia coli* with plasmids. Gene 96:23–28. 10.1016/0378-1119(90)90336-P. 2265755

[48] Wilson DS, Keefe AD. 2001. Random mutagenesis by PCR. Curr Protoc Mol Biol Chapter 8:Unit 8.3. 10.1002/0471142727.mb0803s51. 18265275

[49] Cirino PC, Mayer KM, Umeno D. 2003. Generating mutant libraries using error-prone PCR, p 3–10. *In* Arnold FH, Georgiou G (ed), Directed evolution library creation: methods and protocols. Humana Press, Totowa, NJ. 10.1385/1-59259-395-X:3 12824595

[50] Ambler RP, Coulson AFW, Frère JM, Ghuysen JM, Joris B, Forsman M, Levesque RC, Tiraby G, Waley SG. 1991. A standard numbering scheme for the class A beta-lactamases. Biochem J 276:269–272. 10.1042/bj2760269. 2039479 PMC1151176

[51] Newman J, Egan D, Walter TS, Meged R, Berry I, Ben Jelloul M, Sussman JL, Stuart DI, Perrakis A. 2005. Towards rationalization of crystallization screening for small- to medium-sized academic laboratories: the PACT/JCSG+ strategy. Acta Crystallogr D Biol Crystallogr 61:1426–1431. 10.1107/S0907444905024984. 16204897

[52] Kabsch W. 2010. XDS. Acta Crystallogr D Biol Crystallogr 66:125–132. 10.1107/S0907444909047337. 20124692 PMC2815665

[53] Evans PR. 2011. An introduction to data reduction: space-group determination, scaling and intensity statistics. Acta Crystallogr D Biol Crystallogr 67:282–292. 10.1107/S090744491003982X. 21460446 PMC3069743

[54] Winn MD, Ballard CC, Cowtan KD, Dodson EJ, Emsley P, Evans PR, Keegan RM, Krissinel EB, Leslie AGW, McCoy A, McNicholas SJ, Murshudov GN, Pannu NS, Potterton EA, Powell HR, Read RJ, Vagin A, Wilson KS. 2011. Overview of the CCP4 suite and current developments. Acta Crystallogr D Biol Crystallogr 67:235–242. 10.1107/S0907444910045749. 21460441 PMC3069738

[55] Joosten RP, Long F, Murshudov GN, Perrakis A. 2014. The PDB-REDO server for macromolecular structure model optimization. IUCrJ 1:213–220. 10.1107/S2052252514009324. PMC4107921 25075342

[56] Sobolev OV, Afonine PV, Moriarty NW, Hekkelman ML, Joosten RP, Perrakis A, Adams PD. 2020. A global Ramachandran score identifies protein structures with unlikely stereochemistry. Structure 28:1249–1258.e2. 10.1016/j.str.2020.08.005. 32857966 PMC7642142

[57] Chen VB, Arendall WB, Headd JJ, Keedy DA, Immormino RM, Kapral GJ, Murray LW, Richardson JS, Richardson DC. 2010. MolProbity: all-atom structure validation for macromolecular crystallography. Acta Crystallogr D Biol Crystallogr 66:12–21. 10.1107/S0907444909042073. 20057044 PMC2803126

[58] Vranken WF, Boucher W, Stevens TJ, Fogh RH, Pajon A, Llinas M, Ulrich EL, Markley JL, Ionides J, Laue ED. 2005. The CCPN data model for NMR spectroscopy: development of a software pipeline. Proteins 59:687–696. 10.1002/prot.20449. 15815974

[59] Vallurupalli P, Hansen DF, Stollar E, Meirovitch E, Kay LE. 2007. Measurement of bond vector orientations in invisible excited states of proteins. Proc Natl Acad Sci U S A 104:18473–18477. 10.1073/pnas.0708296104. 18006656 PMC2141801

[60] Delaglio F, Grzesiek S, Vuister GW, Zhu G, Pfeifer J, Bax A. 1995. NMRPipe: a multidimensional spectral processing system based on UNIX pipes. J Biomol NMR 6:277–293. 10.1007/BF00197809. 8520220

[61] Hansen DF, Vallurupalli P, Lundström P, Neudecker P, Kay LE. 2008. Probing chemical shifts of invisible states of proteins with relaxation dispersion NMR spectroscopy: how well can we do? J Am Chem Soc 130:2667–2675. 10.1021/ja078337p. 18237174

