# Supplementary material for: Two β-Lactamase Variants with Reduced Clavulanic Acid Inhibition Display Different Millisecond Dynamics
Source: Antimicrob Agents Chemother. 2021 Jul 16;65(8):e02628-20. doi: 10.1128/AAC.02628-20 (PMC8284444; doi:10.1128/AAC.02628-20)
Supplement: Supplemental file 1 — Supplemental material. Download AAC02628-20_Supp_S1_seq7.pdf, PDF file, 2.2 MB [file aac02628-20_supp_s1_seq7.pdf]

Supplementary material

to

Two β-lactamase variants with reduced clavulanic acid inhibition display different millisecond dynamics

Wouter Elings, Aleksandra Chikunova, Danny B. van Zanten, Ralphe Drenth, Misbha Ud Din Ahmad, Anneloes J. Blok, Monika Timmer, Anastassis Perrakis and Marcellus Ubbink

SUPPLEMENTARY TEXT

Optimization of the library generation pipeline

Functional screening of a large library of mutant proteins requires a different approach than the overexpression of soluble protein in expression-optimised cell systems such as routinely used for protein production. To target the protein to its functional location in *Escherichia coli*, the periplasm, the gene encoding the soluble domain of BlaC was fused to an N-terminal, TorA-derived protein export signal (Figure S1). To achieve the high transformation efficiencies required for library generation, the small, non-β-lactam-selective plasmid pUK21 was used (Figure S2). The digestion / ligation approach was compared to Gibson Assembly (in-house), the InFusion Cloning Kit (Takara, Inc.) and the GeneMorph® II EZClone Domain Mutagenesis Kit (Agilent Technologies). The in-house digestion / ligation approach was chosen because it outscored the other approaches in the number of transformed mutants per transformation. The most efficient insert : vector molar ratio for ligation and subsequent transformation was found to be 3 or 4 : 1 (Figure S11). *E. coli* strain KA797 was found to be the most efficient strain for transformation that was available in-house. Surprisingly, chemical transformation was found to yield more transformants than did electrotransformation. Commercial XL10-Gold cells were found to be ~3x more competent than KA797 cells when the latter were prepared via the standard in-house chemically competent cell preparation protocol, but an order of magnitude less than KA797 cells prepared via the 'Simple and Efficient Method' (SEM) described by Inoue *et al.*[1] (Table S4). Strain KA797 was also found to be a useful strain for library screening (Table S5). An ampicillin concentration of 8 µg/mL was found to be the minimum required concentration to prevent any non-resistant KA797 cell from forming a colony on the plate after transformation. Likewise, 1 µg/mL clavulanic acid was found to be enough to inhibit colony formation of all wt BlaC expressing cells. When incubated at room temperature (with 1 mM IPTG), KA797 cells that were transformed with the BlaC cloning/expression plasmid were only resistant to ~2 µg/mL ampicillin (*vs.* ~16 µg/mL at 310 K). Pre-induction with IPTG or switching to *E. coli* strain DH5α did not increase this MIC. Screening for mutants with increased clavulanic acid resistance but decreased temperature stability was therefore not attempted.

SUPPLEMENTARY FIGURES

ATG*gccaataatgacctgtttcaggcaagccgtcgtcgttttctggcacagctgggtggtctgaccgttg*
*caggtatgctgggtccgagcctgctgacaccgcgtcgtgcaaccgcagcacaggca*GATCTGGCAGATCG
TTTTGCAGAACTGGAACGTCGTTATGATGCACGTCTGGGTGTTTATGTTCCGGCAACCGGCACCACCGCA
GCAATTGAATATCGTGCAGATGAACGTTTTGCATTTTGCAGCACCTTTAAAGCACCGCTGGTTGCAGCCG
TTCTGCATCAGAATCCGCTGACCCATCTGGATAAACTGATTACCTATACCAGTGATGATATCCGTAGCAT
TAGTCCGGTTGCACAGCAGCATGTTCAGACCGGTATGACCATTGGTCAGCTGTGTGATGCAGCAATTCGT
TATAGTGATGGCACCGCAGCCAATCTGCTGCTGGCCGATCTGGGTGGACCGGGTGGTGGTACAGCAGCCT
TTACCGGTTATCTGCGTAGCCTGGGTGATACCGTTAGCCGTCTGGATGCAGAAGAACCGGAACTGAATCG
TGATCCGCCTGGTGATGAACGTGATACCACCACACCGCATGCCATTGCACTGGTTCTGCAGCAGCTGGTT
CTGGGTAATGCACTGCCTCCGGATAAACGTGCACTGCTGACCGATTGGATGGCACGTAATACCACCGGTG
CCAAACGTATTCGTGCAGGTTTTCCGGCAGATTGGAAAGTTATTGATAAAACCGGTACGGGTGATTATGG
TCGTGCAAATGATATTGCAGTTGTTTGGAGCCCGACCGGTGTTCCGTATGTTGTTGCAGTTATGAGCGAT
CGTGCCGGTGGTGGCTATGATGCCGAACCGCGTGAAGCACTGCTGGCGGAAGCAGCAACCTGTGTTGCCG
GTGTTCTGGCActcgagcaccaccaccaccaccacTGA

Figure S1. Sequence of the BlaC gene that was used for generation of the mutant library. Bases in upper case encode the BlaC protein, lower case italics encodes the TorA-derived protein export signal for use in *E. coli*, lower case without italics encodes a C-terminal 6-histidine purification tag. Start (ATG) and stop (TGA) codons are in capitals.

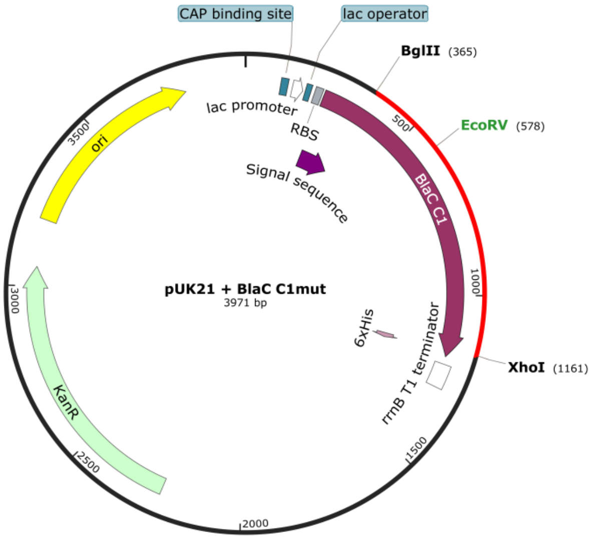

Figure S2. Map of the cloning/expression plasmid used for generation of the mutant library. The section of DNA that was mutated through error-prone PCR is displayed in red.

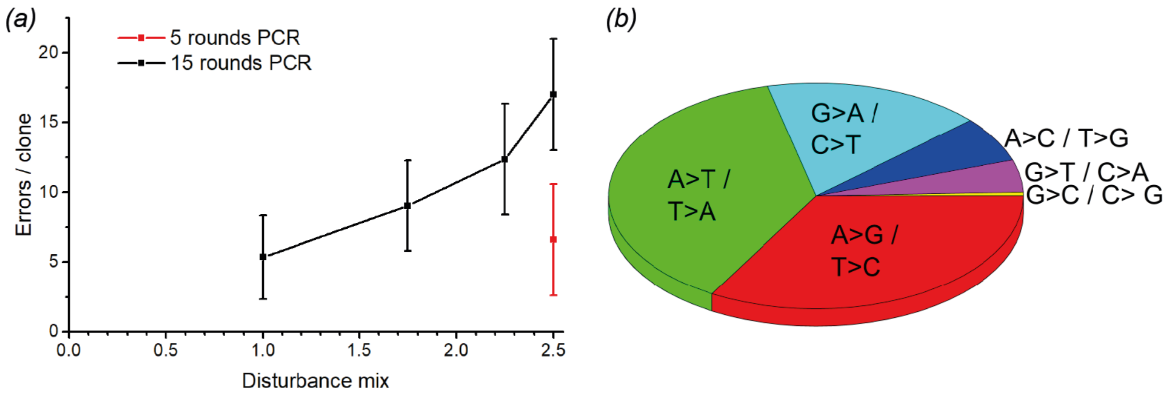

Figure S3. Semi-random mutagenesis. *(a)* Mutation frequency average and standard deviation as a function of the relative concentration of ‘disturbance mix’, with 1.0 indicating 0.2 mM manganese, 2 mM extra magnesium and 0.32 mM extra dCTP/dTTP. Fifteen rounds of PCR with 1x disturbance mix was used for generation of the library. *(b)* Mutation bias, based on 369 single base pair replacements.

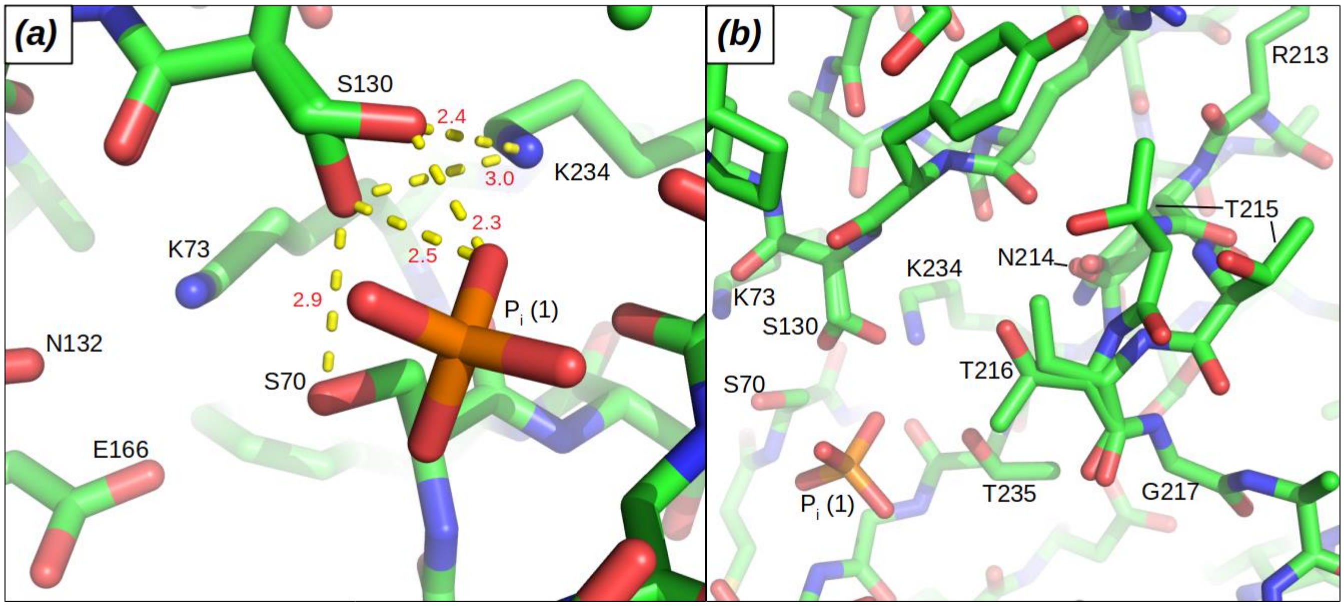

Figure S4. Multiple conformations of Ser130 *(a)* and the Asn214 – Thr216 loop *(b)* in the BlaC G132N crystal structure. Distances between the Ser130 side chain oxygen and nearby heavy atoms are indicated in red, in Å.

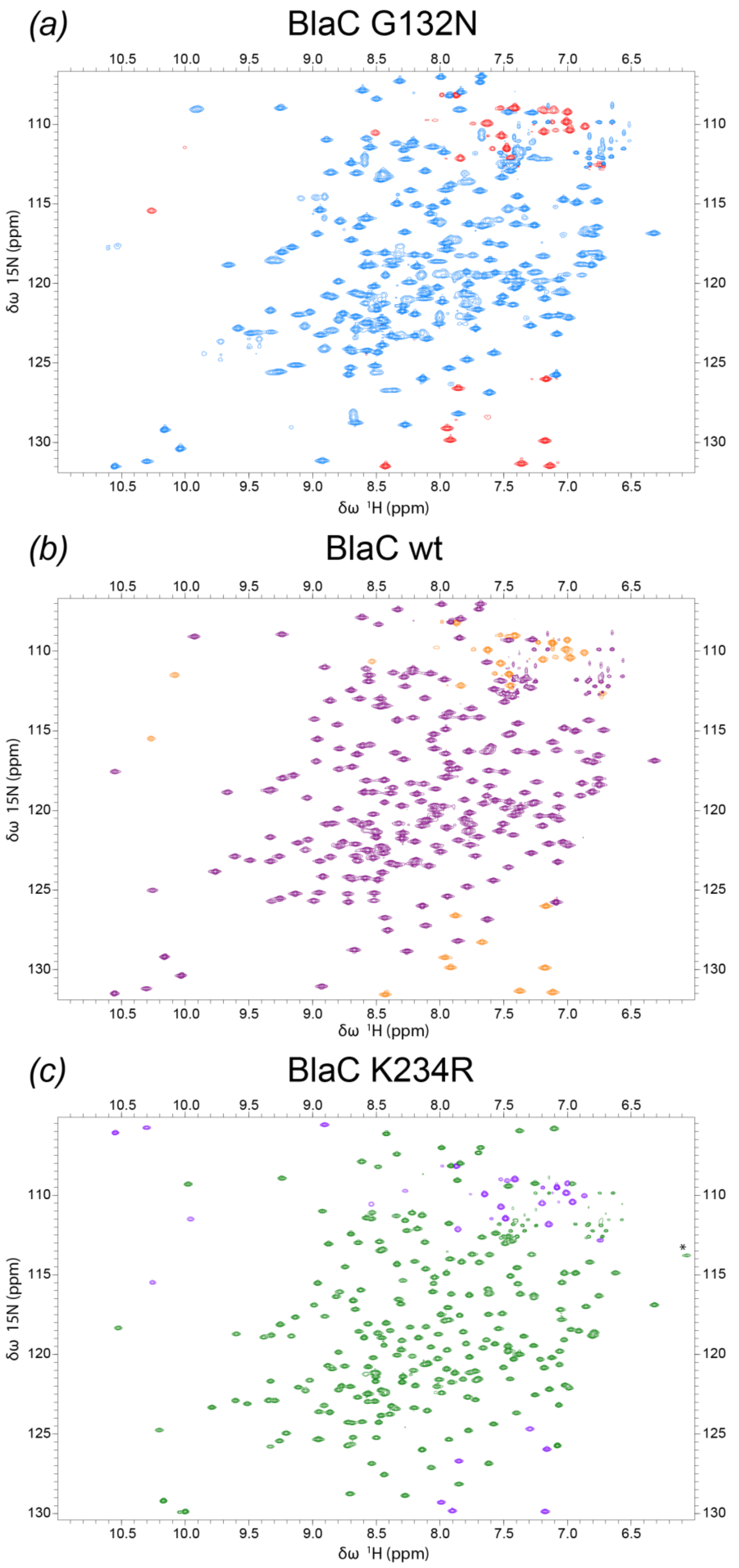

Figure S5. TROSY-HSQC spectra of BlaC wt and mutants. In each case, contour levels in the minority color indicate folded peaks. Assignments have been deposited in the Biological Magnetic Resonance Bank, IDs 27889, 27888 and 27891 for BlaC G132N, wt and K234R, respectively. The resonance marked with an * in the BlaC K234R spectrum is assigned to the Thr235 amide.

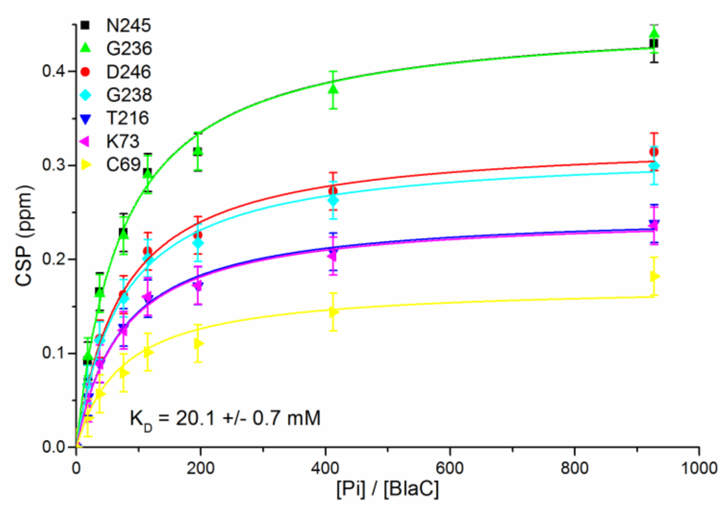

Figure S6. BlaC K234R – phosphate binding curves. The plot shows the CSPs upon phosphate titration for seven selected backbone amide resonances plotted against the ratio of the phosphate and BlaC concentrations. Data points are shown with an estimated peak picking error of ±0.02 ppm, error in K_D is the standard error of the global fit.

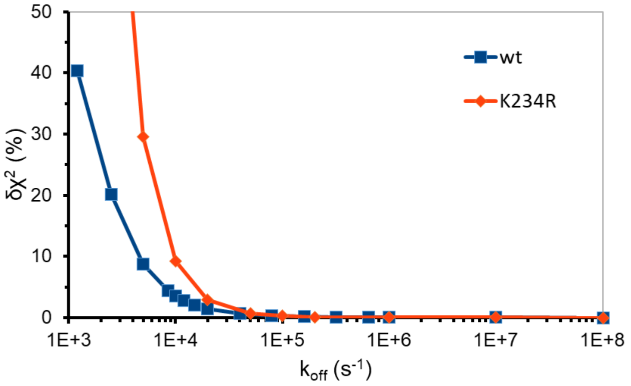

Figure S7. Dependence of phosphate titration fits on off-rate. Regular residuals of best fits to titration data of BlaC wt (blue squares) and K234R (orange diamonds) with phosphate, with fixed off-rate k_{off}, are displayed relative to the best fits with k_{off} not fixed. The wt titration data is from Elings *et al.*[2] A two-state binding model was fitted to the peak shapes in the titration spectra, using the software TITAN.[3] For the wt data, the F72, K73, A74, G132, G236, G238 and D246 backbone amide resonances were used. For the K234R titration data, the C69 backbone amide resonances were used. This plot shows that the data only provide a lower limit estimate of the off-rate.

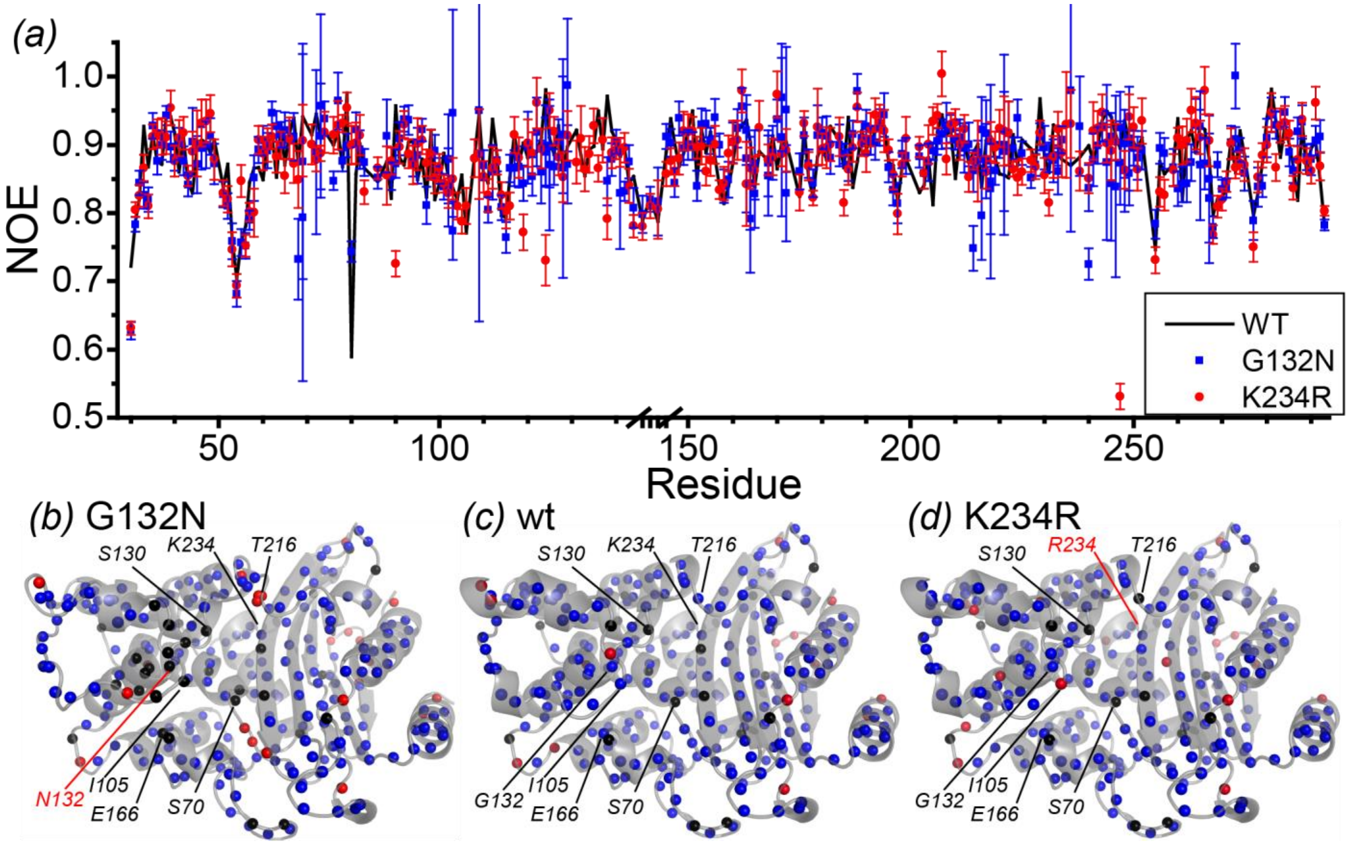

Figure S8. Nuclear Overhauser effects (*NOE*) of backbone amides in wt and mutant BlaC. *(a)* Plot of *NOE* for each residue. The break on the horizontal axis represents the additional BlaC G-G-G-T-loop, relative to Ambler numbering. Error bars indicate the standard deviation based on the spectral noise. The wt BlaC *NOE* values are displayed as a black line for reference, values and errors can be found under BMRB ID 27888.[4] *(b-d)* Plots of *NOE* values on the structure of BlaC G132N *(b)*, and on the wt BlaC structure (PDB entry 5NJ2, chain A)[2] for wt *(c)* and K234R *(d)*. Backbone amides with *NOE* < 0.8 are displayed in *red*, those with *NOE* ≥ 0.8 in *blue* and those for which no data are available in *black*.

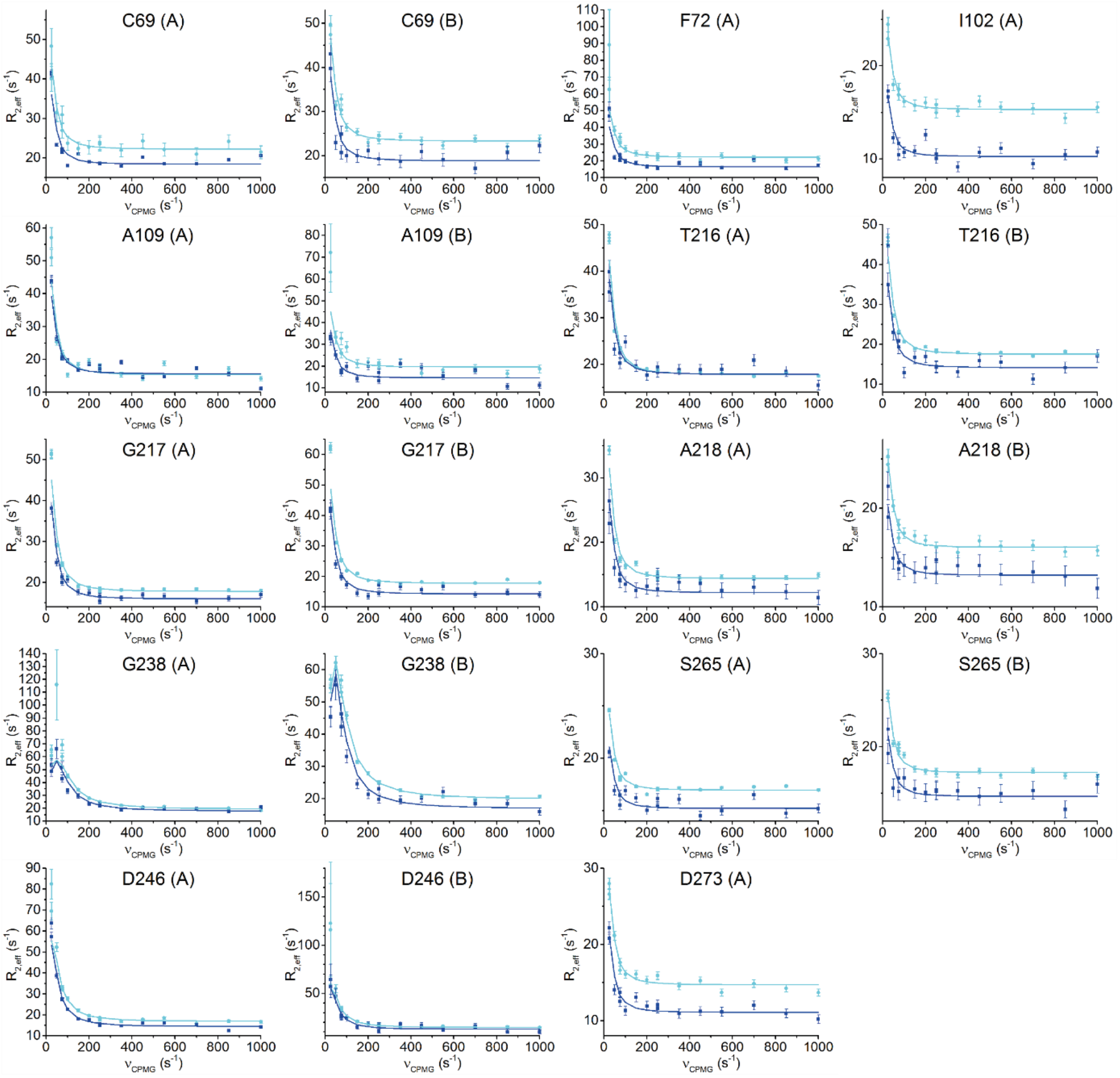

Figure S9. Relaxation dispersion curves of G132N backbone amide resonances. Blue squares and cyan circles represent data obtained at 20.0 and 22.3 T, respectively. Lines indicate grouped two-field fits to the eleven ‘state A’ resonances or to the eight ‘state B’ resonances, yielding exchange rates of 84 ± 3 or 92 ± 3 s^{-1}, respectively. Error bars represent the standard deviation based on three duplicate CPMG frequencies.

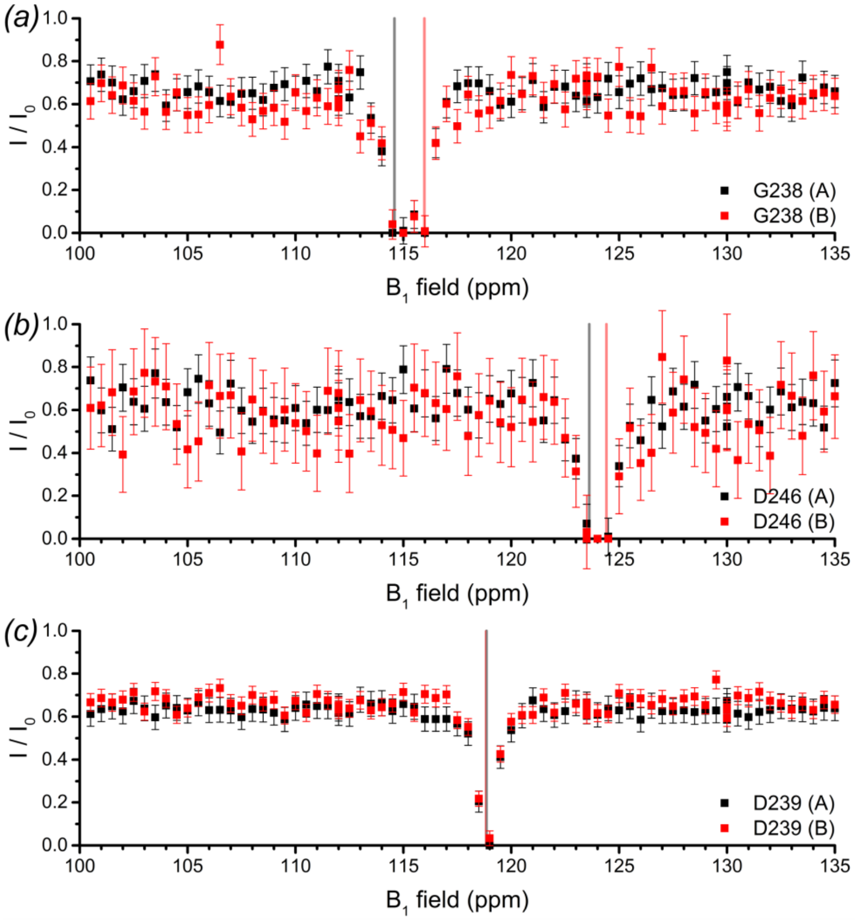

Figure S10. CEST profiles of G132N backbone amides Gly238 *(a)*, Asp246 *(b)* and Asp239 BlaC *(c)*. Vertical bars represent the peak positions of state A (black) and B (red). In panels *(a)* and *(b)*, the ^{15}N chemical shift difference between states A and B is large and exchange between the states can be observed via broadening of the two saturation dips. Panel *(c)* is included for reference, here the observable ^{15}N chemical shift difference between states A and B is small and the exchange is therefore not detectable. All other profiles (not shown) have profiles that are similar to *(c)*. Error bars indicate the standard deviation based on the spectral noise. These errors, especially those in *(b)*, are very large due to the splitting and broadening of the peaks.

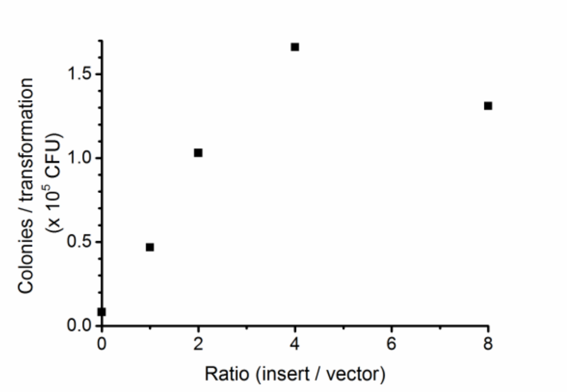

Figure S11. Transformation efficiency as a function of ligation condition.

SUPPLEMENTARY TABLES

Table S1. Primers that were used in this study.[a]

Process	Primer sequences (5' - 3')
Error-prone PCR	GCTAGCTCAGTCCTAGGTATAATGCTAGCGTTTAACTTTAAGAAGGAGATATACCATGG;<br>CCCGGGAGCTCGAATTCCCTCAGTGGTGGTGGTGGTGGTG
Second amplification	CGCCACGGCGATATCGGATCCTTGACAGCTAGCTCAGTCCTAGGTATAATGC;<br>CCCGGGAGCTCGAATTCCC
G132N mutagenesis	GCAGCAATTCGTTATAGTGATAACACCGCAGCCAATCTGCTGC;<br>GCAGCAGATTGGCTGCGGTGTTATCACTATAACGAATTGCTGC
K234R mutagenesis	GCAGATTGGAAAGTTATTGATAGAACCGGTACGGGTGATTATGG;<br>CCATAATCACCCGTACCGGTTCTATCAATAACTTTCCAATCTGC
I105V mutagenesis	GTGATGATATCCGTAGCGTTAGTCCGGTTGCACAGC;<br>GCTGTGCAACCGGACTAACGCTACGGATATCATCAC
R213S mutagenesis	GCTGACCGATTGGATGGCAAGTAATACCACCGGTGCCAAACG;<br>CGTTTGGCACCGGTGGTATTACTTGCCATCCAATCGGTCAGC
H184R mutagenesis	CGTGATACCACCACACCGCGTGCCATTGCACTGGTTCTGC;<br>GCAGAACCAGTGCAATGGCACGCGGTGTGGTGGTATCACG
D172N mutagenesis	GCAGAAGAACCGGAACTGAATCGTAATCCGCCTGGTGATGAACG;<br>CGTTCATCACCAGGCGGATTACGATTCAGTTCCGGTTCTTCTGC

[a] Bases that are displayed in red indicate mutations with respect to the wt sequence.

Table S2. Data collection and refinement statistics for BlaC G132N structure PDB 7A74.

Data Collection	
Wavelength (Å)	0.912
Resolution (Å)	44.99-1.6 (1.63-1.6)
Space group	P 21 21 21
Unit cell a, b, c (Å)	54.00, 54.60, 79.40
$CC_{1/2}$	99.7 (63.0)
R_{pim} (%)	5.6 (39.5)
$\|I/\sigma I\|$	6.0 (1.5)
Completeness (%)	99.0 (93.6)
Multiplicity	1.8
Unique reflections	31258
**Refinement**	
Atoms protein / ligands / water	2106 / 94 / 167
B-factors protein / ligands / water ($Å^2$)	14 / 34 / 28
R_{work} / R_{free} (%)	15.5 / 18.8
Bond lengths RMSZ / RMSD (Å)	0.535 / 0.014
Bond angles RMSZ / RMSD (°)	0.777 / 1.59
Ramachandran preferred / outliers	252 / 2
Clash score	2.5
MolProbity score	1.03

Table S3. CPMG relaxation dispersion data and fits thereof.

Variant	Fields (T)	Peaks used in grouped fit
wt[a]	14.1 & 20.0	F68, C69, T71, E166, E168, N170, T215 and A218
G132N[b]	20.0 & 22.3	State A: C69, F72, I102, A109, T216, G217, A218, G238, S265, D246 and D273
		State B: C69, A109, T216, G217, A218, G238, S265 and D246
K234R[c]	20.0	None

[a] Wild type data and fit are from Elings *et al.*[4]

[b] For G132N, two separate two-field fits were performed; one for each state.

[c] For K234R, no significant relaxation dispersion was detected.

Table S4. Transformation efficiency of SEM competent *E. coli* KA797 as a function of DNA concentration.[a]

Plasmid (ng)	CFU ($\times 10^{4}$)	CFU / Plasmid molecule ($\times 10^{-5}$)	CFU / cell ($\times 10^{-4}$)
0.125	2	70	3
1.25	29	100	40
**12.5**	**142**	**50**	**200**
42.5	177	20	200
85	331	20	500
170	341	9	500
425	461	5	600
850	625	3	800

[a] This test was performed with intact plasmids. The approximate amount of DNA per transformation that was used for the library generation is indicated in bold.

Table S5. Ampicillin minimal inhibitory concentration (MIC) for several *E. coli* strains with and without BlaC.[a]

Strain	Ampicillin MIC (µg/mL)	
	- BlaC	+ BlaC
KA797	2	32
BL21 Codon+	0.5	16
BL21 pLys CPF	0.5	16
BL21 STAR (DE3) pLys	1	32
BL21 pLysE	0.5	0.5
BL21 pLysS	0.5	64

[a] Plates were incubated overnight at 310 K in the presence of 1 mM IPTG, after application of 150 µL of cultures with 30 minutes pre-induction and cell density OD_{600} = 10.

SUPPLEMENTARY REFERENCES

(1) Inoue, H., Nojima, H., and Okayama, H. (1990) High efficiency transformation of *Escherichia coli* with plasmids. *Gene 96*, 23–28.

(2) Elings, W., Tassoni, R., van der Schoot, S. A., Luu, W., Kynast, J. P., Dai, L., Blok, A. J., Timmer, M., Florea, B. I., Pannu, N. S., and Ubbink, M. (2017) Phosphate promotes the recovery of Mycobacterium tuberculosis β-lactamase from clavulanic acid inhibition. *Biochemistry 56*, 6257–6267.

(3) Waudby, C. A., Ramos, A., Cabrita, L. D., and Christodoulou, J. (2016) Two-Dimensional NMR Lineshape Analysis. *Sci. Rep. 6*, 24826.

(4) Elings, W., Gaur, A., Blok, A. J., Timmer, M., van Ingen, H., and Ubbink, M. (2019) β-Lactamase of *Mycobacterium tuberculosis* shows dynamics in the active site that increase upon inhibitor binding. *Antimicrob. Agents Chemother.*
